# Whole genome sequencing identifies monogenic disease in 56.1% of families with early-onset steroid-resistant nephrotic syndrome

**DOI:** 10.1007/s00439-025-02752-y

**Published:** 2025-05-22

**Authors:** Neveen A. Soliman, Mohamed A. Elmonem, Ahmed F. El-Sayed, Eman Ramadan, Ahmed M. Badr, Fatma M. Atia, Rasha Helmy, May O. Amer, Ahmed Abd El-Raouf, Fadya M. El-Garhy, Omnia M. Abdel-Haseb, Tokka M. Hassan, Yasmeen K. Farouk, Ahmed El-Hosseiny, Usama Bakry, Asmaa Ali, Sheri Saleeb, Tasnim A. Ghanim, Mahynour Albarbary, Ahmed Elmahy, Tarek Elnagdy, Amira Ragheb, Wael A. Hassan, Ahmed Moustafa, Khaled Amer

**Affiliations:** 1 Department of Pediatrics, Center of Pediatric Nephrology and Transplantation (CPNT), Faculty of Medicine, Cairo University, Cairo, Egypt; 2 Egypt Center for Research and Regenerative Medicine (ECRRM), Cairo, Egypt; 3 EGORD, Egyptian Group for Orphan Renal Diseases, Cairo, Egypt; 4 Department of Clinical and Chemical Pathology, Faculty of Medicine, Cairo University, Cairo, Egypt; 5 Department of Microbial Genetics, Biotechnology Research Institute, National Research Centre (NRC), Giza, Egypt; 6 Pharmacology and Biochemistry Department, Faculty of Pharmacy, The British University in Egypt, Cairo, Egypt; 7 Department of Biology, American University in Cairo, New Cairo, Egypt

## Abstract

Genetic causes of steroid-resistant-nephrotic-syndrome (SRNS) represent a rapidly growing number of monogenic diseases. The reported diagnostic yield of various studies applying genetic panels and exome-sequencing to diagnose SRNS is usually < 30%. We performed genome-sequencing in a cohort of Egyptian SRNS patients. We recruited 47 SRNS patients belonging to 41 unrelated families [28 males/19 females; median (range): 6 (0.5–22 years)]. We established a pipeline for genome sequencing, bioinformatics analysis, variant curation and protein modeling at the Egypt Center for Research and Regenerative Medicine (ECRRM). Disease-causing variants were detected in 27/47 patients (57.4%) belonging to 23/41 families (56.1%), including nine novel variants in *NPHS1, NPHS2, COL4A3, MYO1E, NUP93, PLCE1, PODXL, SMARCAL1* and *WT1*. Novel variants were confirmed by Sanger sequencing and were segregated in families of affected patients. *NPHS2* was the most common causative gene in 8/23 (34.8%) of confirmed families, followed by *NPHS1*, *WT1*, and *SMARCAL1* in 2/23 families (8.7%) each. All detected missense variants were evaluated through protein modeling and were predicted deleterious. Our study expanded the spectrum of SRNS disease-causing variants and revealed a monogenic cause in 56.1% of investigated families. In our cohort, no deep intronic or regulatory variants were detected by genome-sequencing. Pursuing genetic diagnosis in SRNS patients is crucial to inform clinical decision making, genetic counseling, transplantation strategy and prenatal diagnosis thus improving clinical outcome of affected patients.

**Supplementary Information:**

The online version contains supplementary material available at 10.1007/s00439-025-02752-y.

## Introduction

The worldwide incidence of idiopathic nephrotic syndrome (NS) in children ranges between 2–7 per 100,000 per year, with an approximate prevalence of 16 per 100,000. The majority of cases respond to glucocorticoid therapy, while approximately 15% of patients do not achieve complete remission after 4–6 weeks of steroid therapy and are labeled steroid-resistant nephrotic syndrome (SRNS) (Eddy and Symons 2003).

The main pathophysiological element causing NS is the impaired function of the glomerular filtration barrier, consisting of highly differentiated epithelial cells or podocytes, the glomerular basement membrane and fenestrated endothelium. The normal podocyte structure and function are essential for the maintenance of normal glomerular filtration; injury to these cells plays a central role in both the initiation and progression of proteinuric kidney diseases (Campbell and Tumlin 2018). Recent knowledge about monogenic causes of nephrotic syndrome as a molecular entity has revealed that the different histopathological lesions of the kidney, such as focal segmental glomerulosclerosis (FSGS), minimal change nephropathy or collapsing glomerulopathy are unspecific lesions that show different patterns of podocyte injury rather than representing specific diseases requiring specific therapies. Indeed, these pathological patterns can be associated with the same genetic disease and the same pathological pattern can be seen in many different genetic diseases with different treatment requirements (Kopp et al. 2020a). Pathogenic variants in genes related to podocyte function causing what is currently known as podocytopathies, are detected in approximately 30% of SRNS patients, whereas an unidentified circulating immune factor is thought to be responsible for the etiology in the remaining cases (Trautmann, et al. 2020; Dossier et al. 2016).

Although there are genes that are more commonly reported as causing SRNS, such as *NPHS1*, *NPHS2*, *WT1* and *PLCE1(*Kopp et al. 2020a; Trautmann, et al. 2020), the genetic spectrum of the syndrome is expanding rapidly, and families associated with novel genes are reported on a regular basis (Dawman et al. 2020; Rossanti et al. 2021; Mann et al. 2021; Teng et al. 2021; Mao et al. 2020; Kampf et al. 2019; Hermle et al. 2018; Rao et al. 2017).Currently, based on our literature search, the number of genes linked to SRNS as a monogenic cause of the phenotype, whether isolated or syndromic, is approximating 100 genes (Kopp et al. 2020a; Trautmann, et al. 2020).

The accurate incidence and prevalence rates of SRNS are not known in many parts of the world; however, they are expected to be exceptionally high in regions with high rates of consanguinity, such as the Middle East and North Africa (MENA) region. Consanguineous marriage rates up to 60% in some Arab countries are expected to increase the incidence rates of monogenic recessive disorders (Tadmouri et al. 2009). The rates of consanguineous marriages in Egypt at 35% are not the highest in the region; however, it is high enough to aggravate the overall genetic disease burden considerably, especially since the majority of these marriages are between first cousins (86%) (Shawky et al. 2011).

NS morbidity and mortality are either the consequence of the disease or the use of nonspecific therapeutics. The current classification and treatment of NS relies on histological findings or response to steroid therapy while overlooking the underlying etiology in genetic NS.

Updating NS stratification based on a understanding of its underlying molecular mechanisms better informs clinical decisions to achieve precision in NS management. Confirming a genetic etiology in SRNS patients can end patients’ and families’ diagnostic odysseys, spare patients from the unnecessary aggressive arsenal of immunosuppressive medications and their associated adverse effects and preserve health care system resources. Furthermore, confirmation of the genetic etiology of SRNS has a tremendous impact on the patient management plan, particularly regarding the selection of kidney transplantation donors, recurrence risk and the possibility of preemptive kidney transplantation in CKD patients.

In recently reported studies, the most common approach investigating SRNS genetics is either by using targeted genetic panels or exome sequencing, with a diagnostic success rate ranging between 5 and 30% depending on the sequencing technique and recruitment approach (Xiao and Hildebrandt 2022; Rao et al. 2019; Riedhammer et al. 2020; Schapiro et al. 2019; Sadowski et al. 2015). In this study, we performed genome sequencing for the first time as a first-tier genetic approach in a relatively large cohort of young patients with SRNS in a trial to elucidate their genetic background and guide nephrologists to provide optimal therapeutic care.

## Patients and methods

### Patients

Forty-seven young patients from 41 unrelated Egyptian families with SRNS were enrolled at the Department of Pediatrics, Cairo University Children’s Hospital during the period extending from February 2021 until January 2022. SRNS was defined as the lack of complete remission after four weeks of therapy with prednisone or prednisolone at standard dose (Rovin et al. 2021). Secondary SRNS patients were excluded from the study. Patients were categorized according to the age of onset of nephrotic syndrome into congenital nephrotic syndrome (CNS) presenting within the first three months of life, infantile nephrotic syndrome (INS) presenting between 3 months and one year and childhood onset nephrotic syndrome presenting after one year.

Case notes were reviewed for personal history, age of onset and family history (parental consanguinity and/or affected family member defined as having a history of proteinuria, SRNS or renal dysfunction at a young age). Age of CKD, renal replacement therapy (RRT), if any, and clinical examination with special emphasis on extrarenal manifestations particularly neurological, ocular, auditory, skeletal and urogenital associations were all collected.

Reviewed laboratory investigations included serum albumin, cholesterol, urine analysis, urinary protein/creatinine ratio, serum creatinine and CKD stage (KDIGO 2024) as well as kidney pathology results. Sample analysis procedures, including DNA extraction, genome sequencing, bioinformatics, variant curation and protein modeling, were all performed at the Egypt Center for Research and Regenerative Medicine (ECRRM).

### Genomic DNA extraction, quantification and quality assessment

DNA was extracted from K_2_EDTA blood tubes with the chemagic™ 360 Instrument (2024–0020,Perkin Elmer, Waltham, MA, USA) using the chemagic DNA Blood 400 Kit H96 (CMG-1091, Perkin Elmer) and the 96-chemagic rod head (PN CMG-370, Perkin Elmer). Extraction was performed using 400 µl of blood, and DNA was eluted in 100 µl following the manufacturer’s recommended protocol. Two technical replicates were generated for each biological sample. DNA was quantified by the Qubit 4 Fluorometer (Invitrogen, Carlsbad, CA, USA) using the Qubit 1 × dsDNA High Sensitivity (HS) kit (Q33230, Invitrogen). All samples were checked for impurities using the NanoDrop™ One (Thermo Scientific, Waltham, MA, USA). DNA quality was checked on the LabChip GX Touch (CLS138162, Perkin Elmer) using the Genomic DNA Assay kit (CLS760685, Perkin Elmer) with the DNA Extended Range Chip (CLS138948, Perkin Elmer) following the manufacturer’s recommendations.

### Library preparation and sequencing

Library preparation for genome sequencing was performed using IlluminaTruSeq DNA PCR-free library preparation kit (20,015,962, Illumina) following the manufacturer’s instructions. Samples were normalized to 1 μg, and DNA was sheared by sonication with a focused ultrasonicator M220 (PN 500295, Covaris, Woburn, MA, USA). Sample Purification Beads (15,037,172, Illumina) were used for cleanup and size selection, and adapters were then ligated. Unique indexes were used: IDT for IlluminaTruSeq DNA UD Indexes (20,023,784, Illumina). Fragment sizes and quality for all libraries were measured using the Labchip GX Touch, using the NGS 3 K kit (CLS960013, Perkin Elmer) and the 24 X-Mark LabChip (CLS145331, Perkin Elmer) following the manufacturer’s instructions; the expected fragment size was between 400 and 500 bp. Library quantification was performed using the Qubit 4 Fluorometer. For each sample, 2 µl DNA was taken and added to 198 µl of the Qubit 1X dsDNA Working Solution. Each sample library was normalized to a 4 nM concentration and then to 1 nM, and the libraries were pooled. The 1 nM library pool was spiked with 1% PhiX Control v3 (15,017,397, Illumina). The total library pool was denatured using 0.2 N NaOH for 8 min, followed by a neutralization step with 400 mMTris-HCl pH 8, and finally loaded into the library tube. Sequencing was performed on the IlluminaNovaSeq 6000 (PN 20012850, Illumina) using NovaSeq 6000 S4 Reagent Kit v1.5 (300 cycles) (20,028,312, Illumina). An average of 30 × coverage was obtained for all genomic sequences.

### Bioinformatics analysis

The DNA Seq Pipeline of the DRAGEN Bio-IT Platform https://www.illumina.com/products/by-type/informatics-products/dragen-bio-it-platform.html version 3.9.5 was used with the default parameters to perform the primary analysis of the sequencing data. First, the BCL files were converted to FASTQ files, and then the FASTQ files of each sample were mapped to the human reference genome GRCh38 from (https://genome.ucsc.edu/) and saved in BAM files. The BAM files were used as inputs for the variant calling step to produce the VCF files. The VCF files were annotated using Ensembl Variant Effect Predictor (VEP) version 104 to produce the annotated VCF files (McLaren et al. 2016).

### Variant evaluation

VCF files were processed, and variants were prioritized using BaseSpace Variant Interpreter® (Illumina, San Diego, CA, USA), VarSeq® (Golden Helix, Inc., Bozeman, MT, USA), Congenica (Congenica LTD, Cambridgeshire, United Kingdom) and VarSome® (Lausanne, Switzerland). All genes previously reported as causative of SRNS (**Supplementary Table 1**) including both exonic and intronic regions were evaluated as a first step; however, the whole genome was examined when no conclusive pathogenic/likely pathogenic variants were detected in the target genes. In the second step, we evaluated all pathogenic and likely pathogenic variants detected by the above mentioned software programs across the whole genome and not previously related to SRNS. These variants were evaluated separately in the context of patients'clinical features and through reverse phenotyping if needed and only those overlapping significantly with their corresponding patients'phenotypes were reported. Copy number variants (CNVs) and structural variants (SVs) were assessed using BAM files uploaded to VarSeq® software. The pathogenicity of suspected variants was classified according to the American College of Medical Genetics and Genomics (ACMG) (Richards et al. 2015; Tavtigian et al. 2020) and the Association for Clinical Genomic Science (ACGS) (UK 2023) guidelines. Sanger sequencing was used to confirm the variants revealed by next-generation sequencing and to confirm that novel pathogenic variants properly segregated in parents and available family members according to the reported mode of inheritance for each affected gene.

### Protein structure and modeling

The 3D structure of various proteins harboring missense variants was retrieved from the Protein Data Bank https://www.rcsb.org/. If the experimental structure of a protein did not cover the specific protein domain of interest, an alternative option is to search the AlphaFold protein structure database (Jumper et al. 2021; Varadi et al. 2022). The Human Mutation Analysis (HUMA) database https://huma.rubi.ru.ac.za/ and the web server HUMA-PRIMO were used to generate a 3D structural model for mutant and wild-type proteins and an interactive homology modeling pipeline that can be used to introduce variants into protein structures (Brown and Tastan Bishop 2018). Protein structures were edited, non amino acid elements were removed, and the edited structures were saved in (*.pdb*) format. Structures were then visualized using the UCSF Chimera: (Pettersen et al. 2004). Mutant models were further constructed and analyzed from their wild type using the PyMOL tool: http://pymol.sourceforge.net/ (Bramucci et al. 2012). A protein stability change upon single point mutation was predicted by using MAESTRO web (Laimer et al. 2016) and I-Mutant 2.0 server (Capriotti et al. 2005). Basewise conservation was evaluated by the PhyloP100 score, which evaluates the alignment of the affected base against 100 vertebrate species. The PhyloP100 conservation scores were obtained from the University of California, Santa Cruz (UCSC) genome website (https://genome.ucsc.edu/).

## Results

### Clinical features

Forty-seven SRNS patients (28 males/19 females) belonging to 41 unrelated families were recruited for this study. Their ages ranged from 7 months to 22 years (median age 6 years). Twenty-six families (63.4%) had consanguineous parents, whereas 23 families (56.1%) had more than one affected family member. Patients were categorized according to the age of onset of nephrotic syndrome into 4/47 patients with congenital nephrotic syndrome (CNS) presenting within the first three months of life, 6/47 patients with infantile nephrotic syndrome (INS) presenting between 3 months and one year and 37/47 patients with childhood onset nephrotic syndrome presenting after one year. Extrarenal manifestations were identified in twelve patients (26%) (Table 1).

**Table 1:** Clinical characteristics and outcome of all recruited patients

Patient No	Family No	Age (mo)	Sex	Classification	Consanguinity	Affected family member	Extrarenal manifestations	Kidney pathology	CKD stage at recruitment	RRT	Outcome
1	1	136	F	Childhood	Yes	No	No	MesPGN	G5 A3	HD	living
2	2	12	F	INS	Yes	Yes	No	FSGS	G4 A3	No	Deceased
3	4	48	M	Childhood	Yes	Yes	No	-	G5 A3	HD	Deceased
4	5	40	F	Childhood	Yes	Yes	Skeletal defects	MesPGN	G1 A3	No	Living
5	6	40	F	Childhood	Yes	No	No	FSGS	G1 A3	No	Living
6	7	104	M	Childhood	No	Yes	No	–	G1 A3	No	Living
7		27	F	Childhood	No	Yes	No	–	G1 A3	No	Living
8	8	30	M	Childhood	Yes	Yes	No	–	G1 A3	No	Living
9	9	70	F	Childhood	Yes	No	No	FSGS	G5 A3	No	Living
10	10	25	M	INS	Yes	No	No	–	G1 A3	No	Living
11	11	132	F	Childhood	Yes	No	No	FSGS	G1 A3	No	Living
12	13	18	M	Childhood	Yes	No	Dysmorphism, arachnoid cyst	MCD	G3aA3	PD	Deceased
13	14	7	F	INS	Yes	No	No	MesPGN	G2 A3	No	Living
14	15	96	M	Childhood	No	No	No	MCD	G1 A3	No	Living
15	16	156	M	Childhood	No	No	Learning disability, seizures, skeletal	MCD	G1 A3	No	Living
16	17	11	M	CNS	Yes	Yes	GDD	–	G3aA3	No	Living
17	18	111	M	Childhood	Yes	No	Congenital icthyosis	MCD	G1 A3	No	Living
18	19	84	M	Childhood	No	No	No	FSGS	G3bA3	HD	Deceased
19	20	72	F	Childhood	No	Yes	No	FSGS	G1 A3	No	Living
20	21	108	F	Childhood	No	Yes	Synophrys, hirsutism	FSGS	G1 A3	No	Living
21	22	96	M	Childhood	No	No	No	FSGS	G5 A3	HD	Living
22	23	30	M	INS	Yes	Yes	Fair complexion, blond hair	MCD	G3aA3	No	Living
23		36	M	INS	Yes	Yes	Fair complexion, blond hair	FSGS	G3aA3	HD	Deceased
24	24	101	M	Childhood	Yes	No	Skeletal defects	FSGS	G3aA3	No	Living
25	25	120	M	Childhood	No	No	No	FSGS	G1 A3	No	Living
26	26	38	M	Childhood	Yes	Yes	No	MCD	G1 A3	No	Living
27		84	F	Childhood	Yes	Yes	No	–	G1 A3	No	Living
28	27	252	M	Childhood	Yes	Yes	No	–	G4 A3	KTx	Deceased
29		264	F	Childhood	Yes	Yes	No	–	G1 A3	No	Living
30		192	F	Childhood	Yes	Yes	No	–	G1 A3	No	Living
31	28	12	M	CNS	Yes	no	No	–	G2 A3	No	Living
32	29	204	M	Childhood	No	No	No	FSGS	G5 A3	KTx	Living
33	30	36	M	Childhood	No	Yes	No	MCD	G1 A3	No	Living
34	31	7	M	INS	Yes	Yes	Ambiguous genitalia	–	G3bA3	No	Living
35	32	11	F	CNS	Yes	Yes	Cataract, retinal detachment, GDD	–	G3bA3	No	Deceased
36	33	60	M	Childhood	Yes	No	No	FSGS	G5 A2	KTx	Living
37	34	96	F	CNS	no	No	Hypertrophic cardiomyopathy	FSGS	G5 A3	HD	Living
38	35	38	M	Childhood	No	Yes	No	–	G1 A3	No	Living
39	36	104	M	Childhood	No	Yes	No	–	G2 A3	No	Living
40	37	60	F	Childhood	Yes	Yes	No	–	G1 A3	No	Living
41			M	Childhood	Yes	Yes		– MCD			
42	40	36	M	Childhood	Yes	No	No	MCD	G2 A3	No	Living
43	41	108	F	Childhood	Yes	Yes	No	MCD	G2 A3	No	Living
44	42	51	M	Childhood	No	Yes	No	–	G1 A3	No	Living
45	43	72	F	Childhood	Yes	Yes	No	–	G3aA3	No	Living
46	44	84	F	Childhood	Yes	Yes	No	MCD	G1 A3	HD	Living
47	45	27	M	Childhood	Yes	Yes	No	–	G2 A3	No	Living

CKD: chronic kidney disease; CNS: congenital nephrotic syndrome; GDD: global developmental delay; HD: hemodialysis; INS: infantile nephrotic syndrome; KTx: kidney transplantation; F: female; M: male; Mo: months; PD: peritoneal dialysis. MesPGN: mesangioproliferative glomerulonephritis; MCD: minimal change disease; FSGS: focal segmental glomerulosclerosis.Families 3,12,38 and 39 were not included in this table as they were initially recruited but no samples were available for NGS sequencing

Percutaneous kidney biopsy was performed in 27/47 patients (57.4%). Focal segmental glomerulosclerosis (FSGS) was the most common pathological finding (*n* = 14/27, 51.9%); minimal change disease (MCD) was demonstrated in 10/27(37%), whereas mesangioproliferative glomerulonephritis (MesPGN) accounted for 3/27(11%).

Seven of the study patients (*n* = 7/47, 14.9%) died due to infection or dialysis-related complications (septic shock, pneumonia, peritonitis or intracranial hemorrhage). Of the remaining surviving patients, three patients with advanced CKD had kidney transplantation with good graft function to date.

### Genes and variants detected

We detected 24 variants in SRNS related genes, including 9 novel variants in *NPHS1, NPHS2, COL4 A3, MYO1E, NUP93, PLCE1, PODXL, SMARCAL1* and *WT1* (Table 2). Disease-causing variants in SRNS known genes were detected in 27/47 patients (57.4%) belonging to 23/41 families (56.1%). Detected SRNS-related variants included ten missense variants, five splice-site variants, four frameshift variants, three nonsense variants, one in-frame deletion and one start-loss variant (Table 2). According to the pathogenicity classification, they were ten pathogenic, nine likely pathogenic and five VUS variants. All VUS variants were absent or extremely rare in the gnomAD population database, were strongly predicted by mostonline predictor tools as deleterious and were properly segregating in patients'families. ACMG classifications of all detected SNVs and their justifications are presented in **Supplementary Table 2**.

**Table 2:** Detected variants in SRNS Egyptian families

Family	Gene (Ref. Seq)	Inheritance	Allele 1 (ACMG)	Allele 2 (ACMG)	Exon/intron	Variant type	gnomAD MAF	REVEL	CADD	SpliceAI
1	*MYO1E* (NM_004998.4)	AR	c.1616 + 1G > C (LP)#	c.1616 + 1G > C (LP)#	Intron 15/16	Splice site	0.00			1.0
2	*PLCE1* (NM_016341.4)	AR	c.2779G > T;p(.Gly927 Ter) (LP)#	c.2779G > T;p.(Gly927 Ter) (LP)#	Exon 8	Nonsense	0.00			
4	*SMARCAL1* (NM_014140.4)	AR	c.1096 + 4 A > G (VUS)#	c.1096 + 4 A > G (VUS)#	Intron 5/6	Splice site	0.00			0.79
5	*NPHS2* (NM_014625.4)	AR	c.502 C > T;p.(Arg168 Cys) (P)	c.502 C > T;p.(Arg168 Cys) (P)	Exon 4	Missense	0.00	0.967	32	
6	*NPHS2* (NM_014625.4)	AR	c.890 C > T;p.(Ala297 Val) (P)	c.890 C > T;p.(Ala297 Val) (P)	Exon 8	Missense	0.000007	0.782	27.8	
8	*LMX1B* (NM_001174147.2)	AD	c.737G > A;p.(Arg246Gln) (P)	………………………	Exon 4	Missense	0.00	0.908	28.2	
9	*NPHS2* (NM_014625.4)	AR	c.1 A > T (P)	c.1 A > T (P)	Exon 1	Start Lost	0.00			
10	*NPHS2* (NM_014625.4)	AR	c.467 dup;p.(Leu156PhefsTer11) (P)	c.467 dup;p.(Leu156PhefsTer11) (P)	Exon 4	Frameshift	0.0002			
13	*NUP93* (NM_014669.5)	AR	c.554 A > G;p.(Tyr185 Cys) (VUS)#	c.554 A > G;p.(Tyr185 Cys) (VUS)#	Exon 6	Missense	0.000019	0.782	28	
16	*C12orf57* (NM_138425.4)	AR	c.1 A > G (P)	c.1 A > G (P)	Exon 1	Start Lost	0.000032			
17	*NPHS1* (NM_004646.4)	AR	c.1135 C > T:p.(Arg379 Trp) (LP)	c.1135 C > T:p.(Arg379 Trp) (LP)	Exon 9	Missense	0.000019	0.559	29.4	
18	*ALOX12B* (NM_001139.3)	AR	c.1790 C > A;p.(Ala597Glu) (LP)	c.1790 C > A;p.(Ala597Glu) (LP)	Exon 14	Missense	0.000042	0.974	31	
19	*WT1* (NM_024426.6)	AD	c.700G > C;p.(Gly234 Arg) (VUS)#	………………………	Exon 2	Missense	0.000004	0.685	25.7	
20	*NPHS2* (NM_014625.4)	AR	c.167 del;p.(Glu56GlyfsTer43) (P)	c.167 del;p.(Glu56GlyfsTer43) (P)	Exon 1	Frameshift	0.00			
21	Chr16.11P2 deletion syndrome	AD	16:29,544,981-30189106 del	………………………		CNV				
23a,b	*NPHS2* (NM_014625.4)	AR	c.1 A > T (P)	c.1 A > T (P)	Exon 1	Start Lost	0.00			
24	*SMARCAL1* (NM_014140.4)	AR	c.1860G > A;p.(Trp620 Ter) (LP)	c.1860G > A;p.(Trp620 Ter) (LP)	Exon 12	Nonsense	0.00			
26a,b	*NPHS2* (NM_014625.4)	AR	c.596 dup;p.(Asn199LysfsTer14) (LP)#	c.596 dupA;p.(Asn199LysfsTer14) (LP)#	Exon 5	Frameshift	0.00			
27a,b,c	*NPHS2* (NM_014625.4)	AR	c.934 C > G;p.(Leu312 Val) (LP)	c.934 C > G;p.(Leu312 Val) (LP)	Exon 8	Missense	0.00	0.748	32.2	
28	*NPHS1* (NM_004646.4)	AR	c.2758 T > C;p.(Cys920 Arg) (LP)#	c.3478 C > T;p.(Arg1160 Ter) (LP)	Exon 20, 27	Missense, nonsense	0.00, 0.000099	0.840	25	
31	*WT1* (NM_024426.6)	AD	c.1447 + 5G > A (LP)	………………………	Intron 9/10	Splice site	0.00			0.83
32	*LAMB2* (NM_002292.4)	AR	c.1178_1180 del; p.(Phe393 del)(VUS)	c.1178_1180 del;p.(Phe393 del) (VUS)	Exon 9	In frame del	0.0000051			
33	*PODXL* (NM_001018111.3)	AR	c.1101 + 2 T > C (LP)#	c.1101 + 2 T > C (LP)#	Intron 5/6	Splice site	0.00			0.98
34	*COL4 A3* (NM_000091.5)	AD	c.2126-1G > A (LP)#	………………………	Intron 27/28	Splice site	0.00			1.0
35	*CD2 AP* (NM_012120.3)	AD	c.902 A > T;p.(Lys301Met) (VUS)	………………………	Exon 8	Missense	0.000019	0.798	32	

#, novel variant; LP, likely pathogenic; MAF, minor allele frequency; P, pathogenic; VUS, variant of uncertain significance. The pathogenicity of suspected variants was classified according to the American College of Medical Genetics and Genomics (ACMG) guidelines [21]. Combined annotation-dependent depletion (CADD) and rare exome variant ensemble learner (REVEL) scores for missense variants were obtained from University of California, Santa Cruz (UCSC) website href="https://genome.ucsc.edu/" data-mce-href="https://genome.ucsc.edu/">https://genome.ucsc.edu/, while SpliceAI scores for splicing variants were obtained from href="https://spliceailookup.broadinstitute.org/" data-mce-href="https://spliceailookup.broadinstitute.org/">https://spliceailookup.broadinstitute.org/. All gene symbols and nomenclature were according to the href="https://www.genenames.org/" data-mce-href="https://www.genenames.org/">HUGO Gene Nomenclature Committee (HGNC) website href="https://www.genenames.org/" data-mce-href="https://www.genenames.org/">https://www.genenames.org/. All variant nomenclature complies with the guidelines of the human genome variation society (HGVS) href="http://varnomen.hgvs.org/" data-mce-href="http://varnomen.hgvs.org/">http://varnomen.hgvs.org/. The chosen gene transcript is the most biologically relevant transcript for each gene (MANE-Select transcript) based on the ensembl database href="http://www.ensembl.org/index.html" data-mce-href="http://www.ensembl.org/index.html">http://www.ensembl.org/index.html. The nomenclature of all reported variants has been verified using a variant description validation software ( href="https://variantvalidator.org/" data-mce-href="https://variantvalidator.org/">Mutalyzer 3) href="https://mutalyzer.nl/batchprocessor" data-mce-href="https://mutalyzer.nl/batchprocessor">https://mutalyzer.nl/batchprocessor

**Fig. 1:**
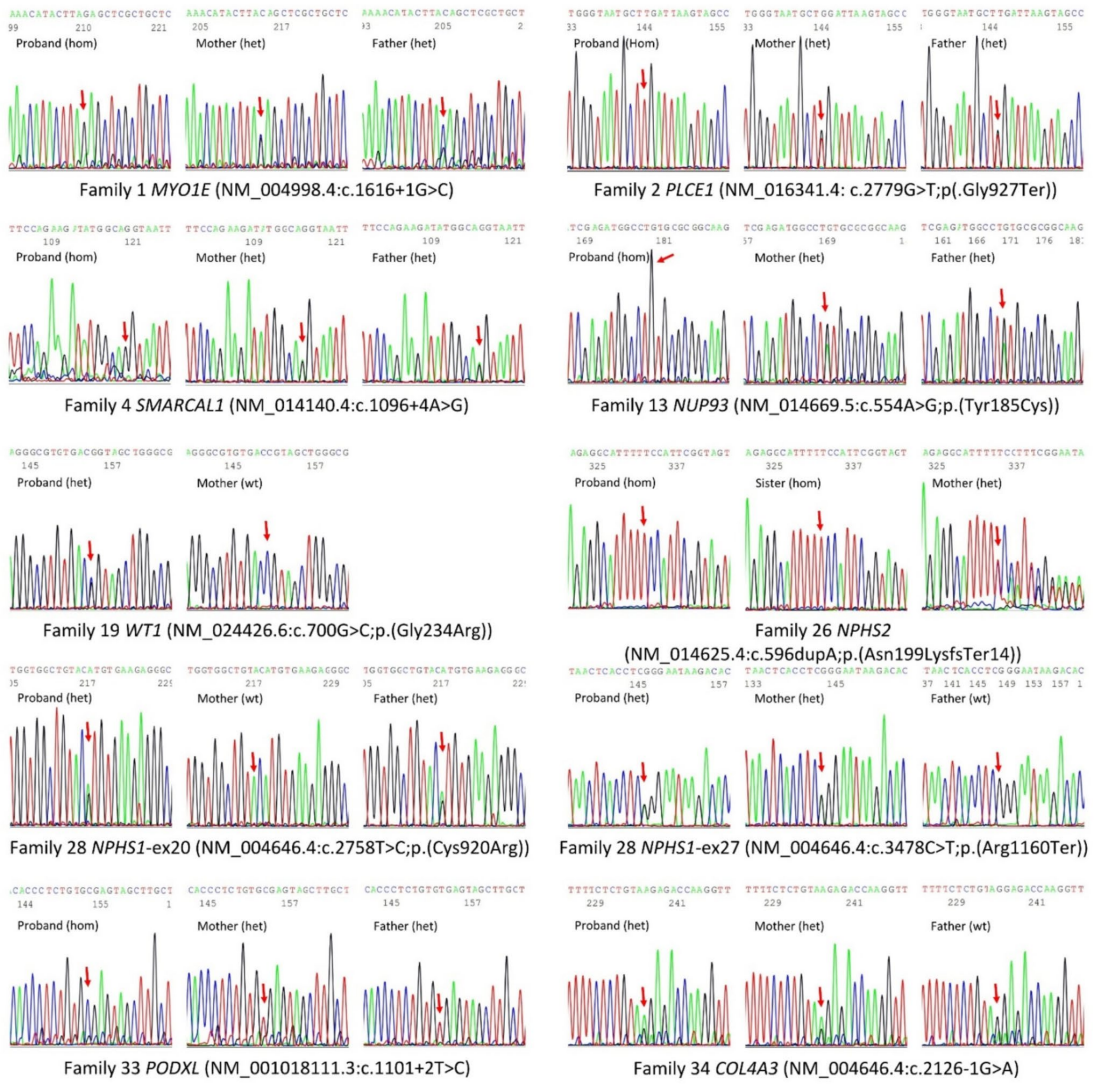
Representation of family segregation by Sanger sequencing for novel variants detected in the study. Both parents were available for sequencing in all families except two families: the father of family 19, who passed away and had a history of chronic renal failure and renal transplantation, and the father of family 26, who was healthy but unavailable for sampling. Red arrows indicate the base change location. Het, heterozygous; hom, homozygous. Variants in *MYO1E* (family 1), *WT1* (family 19), *NPHS2* (family 26) and *NPHS1* (family 28) are sequenced in the reverse strand

All detected novel variants were tested by targeted Sanger sequencing and were confirmed to be segregating properly in families of each proband according to the mode of inheritance of each gene (Fig. 1). All novel variants reported in this study have been submitted to the Leiden Open Variation Database (LOVD) under variant submission codes: #0000972092 (*MYO1E* (NM_004998.4) c.1616 + 1G > C), #0000972093 (*PLCE1* (NM_016341.4)c.2779G > T;p.(Gly927 Ter)), #0000972094 (*SMARCAL1* (NM_014140.4)c.1096 + 4 A > G), #0000972095 (*NUP93* (NM_014669.5) c.554 A > G;p.(Tyr185 Cys)), #0000972096 (*WT1* (NM_024426.6)c.700G > C;p.(Gly234 Arg)), #0000972097 (*NPHS2* (NM_014625.4)c.596 dup;p.(Asn199LysfsTer14)), #0000972098 (*NPHS1* (NM_004646.4) c.2758 T > C;p.(Cys920 Arg)), #0000972099 (*PODXL* (NM_001018111.3)c.1101 + 2 T > C), and #0000972100 (*COL4 A3* (NM_000091.5)c.2126-1G > A).

#, novel variant; LP, likely pathogenic; MAF, minor allele frequency; P, pathogenic; VUS, variant of uncertain significance. The pathogenicity of suspected variants was classified according to the American College of Medical Genetics and Genomics (ACMG) guidelines (Rovin et al. 2021). Combined annotation-dependent depletion (CADD) and rare exome variant ensemble learner (REVEL) scores for missense variants were obtained from University of California, Santa Cruz (UCSC) website https://genome.ucsc.edu/, while SpliceAI scores for splicing variants were obtained from https://spliceailookup.broadinstitute.org/. All gene symbols and nomenclature were according to the HUGO Gene Nomenclature Committee (HGNC) websitehttps://www.genenames.org/. All variant nomenclature complies with the guidelines of the human genome variation society (HGVS) http://varnomen.hgvs.org/. The chosen gene transcript is the most biologically relevant transcript for each gene (MANE-Select transcript) based on the ensembl database http://www.ensembl.org/index.html. The nomenclature of all reported variants has been verified using a variant description validation software (Mutalyzer 3) https://mutalyzer.nl/batchprocessor.

*NPHS2* was the most commonly affected gene in Egyptian SRNS patients, as its variants were detected in 8/23 (34.8%) of genetically confirmed families. This was followed by *NPHS1*, *WT1*, and *SMARCAL1,* each with two affected families (Table 2). No de novo variants were detected in our cohort.

In two other families, we detected pathogenic variants in genes or regions not previously linked to kidney disease but with strong clinical and genetic evidence to be associated with patients’ phenotypes. In family 16, we detected a previously reported homozygous start-loss variant in the *C12orf57* gene (NM_138425.4;c.1 A > G) causing the autosomal recessive Temtamy syndrome. This patient had neurological manifestations and global developmental delay with skeletal deformities consistent with Temtamy syndrome and the variant segregated in his family. In family 21, we detected a heterozygous Chr16p11.2 deletion (**Supplementary Fig. 1**) leading to the loss of heterozygosity of 29 coding genes in chromosome 16 (**Supplementary Table 3**), which is strongly predicted to be pathogenic. The variants in these two families could be incidental findings; however, we cannot exclude the potential of these syndromes contributing to the nephrotic syndrome phenotype.

**Fig. 2:**
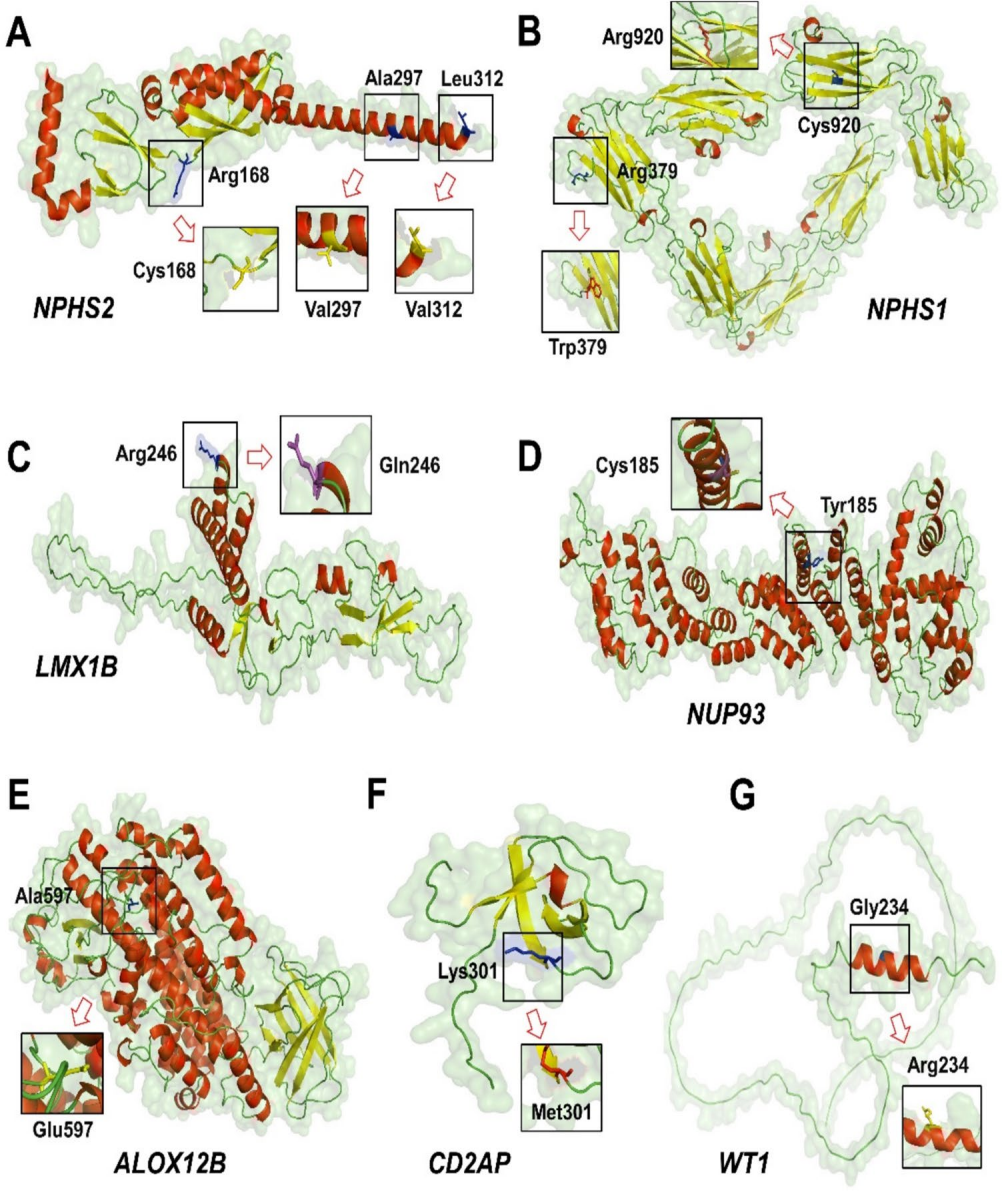
Representation of protein modeling AA changes of the ten detected missense variants in seven genes in the study. **A** *NPHS2*, **B** *NPHS1*, **C** *LMX1B*, **D** *NUP93*, **E** *ALOX12B*, **F** *CD2 AP*, **G** *WT1.*This figure illustrates the 3D protein models highlighting the structural impact of missense variants identified in the study. Each panel **A**–**G** corresponds to a specific gene and its associated variant(s), with key amino acid residues labeled to show their positions and interactions within the protein structure. Red and yellow ribbons represent the protein backbone, while green and white surfaces indicate the surrounding protein environment. Blue and purple sticks highlight the side chains of the mutated residues and their interacting partners. Arrows point to the specific amino acid changes, with zoomed-in insets providing a closer view of the local structural environment. These interactions are critical because changes in amino acid properties such as charge, size, or hydrophobicity can disrupt local protein folding, stability, or binding interfaces. For example: *NPHS2* (Panel A): The p.(Arg168 Cys) substitution replaces a positively charged arginine with cysteine, potentially disrupting ionic interactions or forming aberrant disulfide bonds (Cys168). *NUP93* (Panel D): The p.(Tyr185 Cys) variant replaces a bulky tyrosine with cysteine, likely perturbing the hydrophobic core or introducing a disulfide bond (inset). *CD2 AP* (Panel F): p.(Lys301Met) replaces a positively charged lysine with methionine, potentially impairing electrostatic interactions critical for cytoskeletal binding. *WT1* (Panel G): p.(Glu234 Arg) inverts charge in a zinc-finger DNA-binding domain, suggesting altered transcriptional activity

All missense variants detected in our cohort (ten variants in seven genes) were further evaluated through protein modeling (Fig. 2) using the scores derived from the I-Mutant and MAESTRO software servers to evaluate the amino acid altering effects on protein stability and function and the PhyloP100 score for evaluation of the base conservation at the variant location. The majority of tested missense variants had potentially pathogenic values in all three scores (7/10, 70%), while the remaining three had pathogenic scores in two out of three. Quantitative values and further details are provided in **Supplementary Table 4**.

We further evaluated genotypic-phenotypic correlations that could be detected in our cohort, especially concerning age of disease onset, renal replacement and the presence of extrarenal manifestations. A successful diagnosis of an SRNS related gene was detected at higher rates in lower age groups in association with congenital onset (3/4 families; 75%) and infantile onset (4/5 families; 80%) compared to childhood onset nephrotic syndrome (16/32 families; 50%), although it didn’t reach statistical significance P = 0.257. Three genes were associated with congenital nephrotic syndrome: *NPHS1* (2/2 families), *LAMB2* (1/1 family) and *COL4 A3* (1/1 family). Two genes were associated with infantile onset, *NPHS2* (3/8 families) and *PLCE1* (1/1 family), while the remainder of the diagnosed families presented with childhood onset. Of the 27 patients with performed kidney pathology, a genetic diagnosis was reached in 16/27 (59.2%) with no significant differences among the pathological entities detected: FSGS 9/14 (64.3%), MCD 5/10 (50%) and MesPGN 2/3 (67%) (P = 0.752).

Eight SRNS patients underwent RRT at the time of recruitment (five were on hemodialysis, one was on peritoneal dialysis and two already underwent kidney transplantation). The onset of renal replacement was mainly dependent on the onset of manifestations and was not linked to a specific gene or a specific syndrome. Naturally, the limited number of families affected by each gene restricted our analysis to genotype–phenotype correlations. Nevertheless, when we categorized our diagnosed patients with small variants into two groups with either truncating (15 patients) or in-frame variants (12 patients), the group with truncating variants had significantly lower age (56 ± 41 vs 101 ± 53 months, P = 0.016) and higher CKD stage at recruitment (median 3 vs 1, P = 0.046).

## Discussion

In this study, we investigated the genetic landscape of steroid resistant nephrotic syndrome for the first time in an African or a Middle Eastern country. We used genome sequencing as a first-tier diagnostic test and implied different bioinformatics and variant interpreting tools to reach a plausible diagnosis in almost 56.1% of recruited families. Genetic analysis in SRNS may provide patients and families with a definitive molecular diagnosis, alleviates the burden of unnecessary use of immunosuppressive therapy in genetically confirmed patients, guides proper genetic counseling in the family and population and finally may detect a genotype that can be cured, such as coenzyme Q2 or coenzyme Q10, causing SRNS (Trautmann, et al. 2020; Starr et al. 2018; Tan and Airik 2021). Using exome sequencing, currently < 30% of children are routinely diagnosed with a monogenic disease for SRNS (Bierzynska, et al. 2017), and the likelihood of identifying a causative variant is commonly inversely related to age at disease onset and is increased with familial clustering and/or extra-renal involvement (Trautmann et al. 2015).

In our SRNS cohort, we performed genome sequencing as a first-line approach for the first time worldwide in a relatively large cohort. *NPHS2* gene variants were the most commonly detected etiology in our cohort, constituting approximately 35% of confirmed families. Although the exact prevalence of *NPHS2* variants among Egyptian SRNS patients is largely unknown, several studies from Egypt have previously reported patients with *NPHS2* variants (Thomas et al. 2018, 2022; Bakr et al. 2008; Elshafey et al. 2023). The only variant in our cohort reported in two different families was the start loss *NPHS2* variant: NM_014625.4;c.1 A > T reported in families 9 and 23. This variant has also been reported in four different Egyptian families by (Elshafey et al. 2023), and could have a founder effect in the Egyptian population (Sadowski et al. 2015; Elshafey et al. 2023).

In our cohort, some of the genetic diagnoses were not expected based on the clinical phenotypes and age of onset as in the case of families 32 where *LAMB2* variant was detected as a cause of the congenital nephrotic syndrome (CNS). Although rarely encountered this has been occasionally described in CNS patients (Chen et al. 2011; Zenker et al. 2004).

We confirmed a monogenic SRNS related diagnosis in 27 patients belonging to 23 unrelated families (56.1% of families in the cohort) by detecting variants in 14 different genes. This detection rate is much higher than the average for the reported diagnostic outcome in similar cohorts of young nephrotic syndrome patients. This high diagnostic yield in our study can be explained by many factors, including the high consanguinity rate in the Egyptian population, which facilitates the recurrence of disease-causing variants in the same family and in closed communities. Another factor was the choice of genome sequencing as a first tier test, making the identification of CNVs a much easier task. Finally, we processed our raw data with multiple evaluation software, diminishing the chance of missing a probable disease-causing variant using single software.

Despite the very good diagnostic yield obtained by genome sequencing in our highly consanguineous population, genome sequencing did not detect deep intronic or regulatory variants in our cohort, which is considered a major advantage of such a technique over the much less expensive exome sequencing. We performed a specific search in unresolved families looking for previously reported or potentially novel deep-intronic variants in the genes most commonly associated with SRNS; however, we could not find solid variants to report. Nevertheless, deep intronic variants were reported on multiple occasions in SRNS patients in several genes, including *NPHS2* (Dirix et al. 2023), *NUP93* (Rossanti et al. 2019), and *PLCE1* (Lovric, et al. 2014). Another advantage of both genome and exome sequencing as opposed to targeted gene panels is the potential to detect new genotype–phenotype correlations or the possibility of detecting a clinically relevant genotype that may or may not be directly related to the original phenotype but can alter the patient's life or his management plan drastically as in the case of families 16 and 21 with a detected pathogenic variant in *C12orf57 *gene causing Temtamy syndrome and the Chr16.11P2 deletion syndrome, respectively. Furthermore, the availability of the raw data of the genome sequencing allows for the potential revisiting after a certain period of time when new genes or new variants are reported and confirmed as disease causing for the target phenotype.

Although five of our reported families were diagnosed with variants of uncertain significance in the genes *SMARCAL1, NUP93, WT1, LAMB2* and *CD2 AP* (families 4, 13, 19, 32 and 35, respectively), we considered these variants as the most probable cause of their patients'phenotypes as they are segregating properly in their affected families according to each disease inheritance pattern and they further have many supporting pathogenic criteria, such as being absent or very rare in population databases and being strongly predicted as deleterious by various pathogenicity software.

Identification of a monogenic cause of SRNS has significant implications on patient management through optimizing therapeutic decision-making as it (i) spares the unfavorable side effects of unnecessary immunosuppressive medications (Kopp et al. 2020a; Kopp et al. 2020b; Trautmann et al. 2015); (ii) identifies the disease-causing variant in single gene disorders amenable to therapy (gene of coenzyme Q10 biosynthesis where coenzyme Q10 therapy may be indicated) (Heeringa et al. 2011; Atmaca et al. 2017); (iii) dictates the screening for extrarenal involvement, e.g., surgical evaluation and removal of streak gonads in SRNS patients with identified *WT1* disease-causing variants (Kopp et al. 2020b); (iv) offers the opportunity for early diagnosis and the selection of optimal therapy through screening of at-risk family members, should they opt for it; and (v) allows genetically characterized patients to join clinical trials for novel therapeutics based on molecular stratification of SRNS patients.

Implications of monogenic diagnosis of SRNS extend to kidney transplantation in end-stage kidney disease patients. It allows the reduction of pre- and posttransplant immunosuppression and minimizes the risk of infectious complications, as genetic forms of SRNS are unlikely to recur. Moreover, it allows the utilization of genetic testing in potential living kidney donors and the disqualification of parents or family members testing positive for the disease-causing variant in autosomal dominant monogenic forms of SRNS (e.g., *COL4A3*, *WT1*, *LMX1B* and *CD2AP*). Genomic knowledge is rapidly evolving, thus highlighting the need for genomically oriented nephrologists and nephrogeneticists to keep pace with the latest advances in this energetically dynamic field of genomic medicine. This should improve the ability to integrate genomic and clinical data into nephrology practice thus informing clinical decision-making with substantial impact on the management of SRNS and chronic kidney disease patients (Pinto et al. 2021).

In conclusion, this is the first study implementing genome sequencing as a first-tier test for the diagnosis of SRNS in a large cohort of young Egyptian patients. Our multistep/multievaluation platform approach elucidated a plausible diagnosis in 56.1% of the investigated families. Our study further detected pathogenic variants in genes (*C12orf57*) and regions (Chr16.11P2) that were not previously linked to kidney diseases, which may play a role in the nephrotic syndrome phenotype if other similar families are reported in the future. The identification of monogenic SRNS is fundamental to clinical decision-making. It precludes unnecessary and invasive diagnostics, tailors therapeutic plans, optimizes transplantation approaches and suggests early screening for associated extra-renal disorders while rationalizing health expenditures. The results of the current study enabled genetic counseling and prenatal diagnosis for all genetically confirmed families and can plant the seed of the first national registry for genetic podocytopathies in Egypt.

## Supplementary Information

Below is the link to the electronic supplementary material.

Supplementary file1 (DOCX 1007 KB)

Supplementary file2 (DOCX 222 KB)

## Data Availability

All data generated or analyzed during this study are included in the published article (and its supplementary information files). Any further clinical or genetic data are available from the corresponding author upon reasonable request. All novel variants reported in this study have been submitted to the Leiden Open Variation Database (LOVD) under variant submission codes: #0000972092 to #0000972100.
