# Supplementary material for: Whole genome sequencing identifies monogenic disease in 56.1% of families with early-onset steroid-resistant nephrotic syndrome
Source: Hum Genet. 2025 May 22;144(7):727–40. doi: 10.1007/s00439-025-02752-y (PMC12222417; doi:10.1007/s00439-025-02752-y)
Supplement: Supplementary file 1 — Supplementary file1 (DOCX 1007 KB) [file 439_2025_2752_MOESM1_ESM.docx]

**Whole genome sequencing identifies monogenic disease in 56.1% of families with early onset steroid-resistant nephrotic syndrome**

**Running title: Genomics of SRNS in Egypt**

**Neveen A. Soliman^1,2,3^*^#^, Mohamed A. Elmonem^2,4#^, Ahmed F. El-Sayed^2,5^, Eman Ramadan^2,6^, Ahmed M. Badr^1,3^, Fatma M. Atia^1,3^, Rasha Helmy^1,3^, May O. Amer^2^, Ahmed Abd El-Raouf^2^, Fadya M. El-Garhy^2^, Omnia M. Abdel-Haseb^2^, Tokka M. Hassan^2^, Yasmeen K. Farouk^2^, Ahmed El-Hosseiny^2,7^, Usama Bakry^2^, Asmaa Ali^2^, Sheri Saleeb^2^, Tasnim A. Ghanim^2^, Mahynour Albarbary^2^, Ahmed Elmahy^2^, Tarek Elnagdy^2^, Amira Ragheb^2^, Wael A. Hassan^2^, Ahmed Moustafa^2,7^, Khaled Amer^2^***

^1^Department of Pediatrics, Center for Pediatric Nephrology and Transplantation (CPNT), Faculty of Medicine, Cairo University, Cairo, Egypt

^2^ Egypt Center for Research and Regenerative Medicine (ECRRM), Cairo, Egypt

^3^ EGORD, Egyptian group of Orphan Renal Diseases, Cairo, Egypt

^4^ Department of Clinical and Chemical Pathology, Faculty of Medicine, Cairo University, Cairo, Egypt

^5^ Department of Microbial Genetics, Biotechnology Research Institute, National Research Centre (NRC), Giza, Egypt

^6^ Pharmacology and Biochemistry Department, Faculty of Pharmacy, the British University in Egypt, Cairo, Egypt

^7^ Department of Biology, American University in Cairo, New Cairo, Egypt

***Corresponding authors**

Prof. Dr. Neveen A. Soliman,[nsoliman@kasralainy.edu.eg](mailto:nsoliman@kasralainy.edu.eg); [neveenase@yahoo.com](mailto:neveenase@yahoo.com), ORCID: [0000-0002-8942-1973](https://orcid.org/0000-0002-8942-1973)

Prof. Dr. Khaled Amer, [khaled.amer@ecrrm.ac.eg](mailto:khaled.amer@ecrrm.ac.eg); [dramertx@gmail.com](mailto:dramertx@gmail.com), ORCID 0000-0001-6896-9449

# Authors contributing equally to the study

**Supplementary materials:**

**Supplementary Figure 1.** A diagrammatic representation of the large deletion affecting family 21 (Chr16.11P2 deletion syndrome). Upper panel shows region with the decreased signal compared to the remaining of chromosome 16: Chr16:29544981-30189106del (red arrow). Lower panel shows the multiple reporting of this region as disease causing in ClinVar. The detected CNV is affecting 29 coding genes displayed in Supplementary Table 3.

**Supplementary Table 1.** SRNS related genes used as a virtual gene panel for the initial evaluation of patients.

**Supplementary Table 2.** ACMG criteria for all detected SNVs in Egyptian SRNS patients.

**Supplementary Table 3.** Genes deleted in family 21 with 16.11P2 deletion syndrome.

**Supplementary Table 4.** Evaluation of the effects of protein amino acid changes in detected missense variants on protein structure and function.

**Supplementary Figure 1**

**
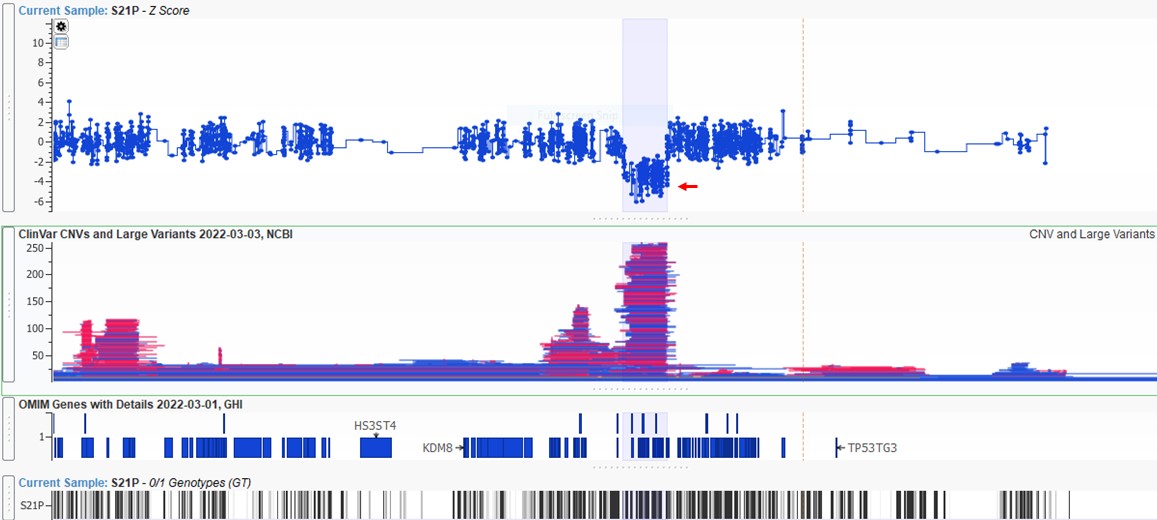
**

**Supplementary Table 1:** SRNS related genes used for the initial evaluation of patients.

| **N** | **Gene** | **Ref-Seq** | **Genomic coordinates (GRCh38)** |
| --- | --- | --- | --- |
|  |  |  | Chromosome:start-end |
| 1 | *ACTN4* | NM_004924.5 | [19:38,647,649-38,731,589](https://genome.ucsc.edu/cgi-bin/hgTracks?db=hg38&position=chr19:38647649-38731589&dgv=pack&knownGene=pack&omimGene=pack) |
| 2 | *ALG1* | NM_019109.4 | [16:5,071,843-5,087,379](https://genome.ucsc.edu/cgi-bin/hgTracks?db=hg38&position=chr16:5071843-5087379&dgv=pack&knownGene=pack&omimGene=pack) |
| 3 | *ALMS1* | NM_015120.4 | [2:73,385,758-73,609,919](https://genome.ucsc.edu/cgi-bin/hgTracks?db=hg38&position=chr2:73385758-73609919&dgv=pack&knownGene=pack&omimGene=pack) |
| 4 | *ALOX12B* | [NM_001139.3](http://www.ncbi.nlm.nih.gov/nuccore/NM_001139.3) | [17:8,072,636-8,087,716](https://genome.ucsc.edu/cgi-bin/hgTracks?db=hg38&position=chr17:8072636-8087716&dgv=pack&knownGene=pack&omimGene=pack) |
| 5 | *ANKFY1* | [NM_001330063.2](http://www.ncbi.nlm.nih.gov/nuccore/NM_001330063.2) | [17:4,163,821-4,263,979](https://genome.ucsc.edu/cgi-bin/hgTracks?db=hg38&position=chr17:4163821-4263979&dgv=pack&knownGene=pack&omimGene=pack) |
| 6 | *ANKS6* | NM_173551.4 | [9:98,732,009-98,796,555](https://genome.ucsc.edu/cgi-bin/hgTracks?db=hg38&position=chr9:98732009-98796555&dgv=pack&knownGene=pack&omimGene=pack) |
| 7 | *ANLN* | NM_018685.4 | [7:36,389,862-36,453,791](https://genome.ucsc.edu/cgi-bin/hgTracks?db=hg38&position=chr7:36389862-36453791&dgv=pack&knownGene=pack&omimGene=pack) |
| 8 | *APOL1* | [NM_003661.4](http://www.ncbi.nlm.nih.gov/nuccore/NM_003661.4) | [22:36,253,133-36,267,525](https://genome.ucsc.edu/cgi-bin/hgTracks?db=hg38&position=chr22:36253133-36267525&dgv=pack&knownGene=pack&omimGene=pack) |
| 9 | *ARHGAP24* | NM_001025616.2 | [4:85,475,150-86,002,666](https://genome.ucsc.edu/cgi-bin/hgTracks?db=hg38&position=chr4:85475150-86002666&dgv=pack&knownGene=pack&omimGene=pack) |
| 10 | *ARHGDIA* | NM_001185077.2 | [17:81,867,721-81,871,337](https://genome.ucsc.edu/cgi-bin/hgTracks?db=hg38&position=chr17:81867721-81871337&dgv=pack&knownGene=pack&omimGene=pack) |
| 11 | *AVIL* | [NM_006576.4](http://www.ncbi.nlm.nih.gov/nuccore/NM_006576.4) | [12:57,797,380-57,818,734](https://genome.ucsc.edu/cgi-bin/hgTracks?db=hg38&position=chr12:57797380-57818734&dgv=pack&knownGene=pack&omimGene=pack) |
| 12 | *CD151* | NM_004357.4 | [11:832,952-838,831](https://genome.ucsc.edu/cgi-bin/hgTracks?db=hg38&position=chr11:832952-838831&dgv=pack&knownGene=pack&omimGene=pack) |
| 13 | *CD2AP* | NM_012120.2 | [6:47,477,789-47,627,263](https://genome.ucsc.edu/cgi-bin/hgTracks?db=hg38&position=chr6:47477789-47627263&dgv=pack&knownGene=pack&omimGene=pack) |
| 14 | *CFH* | NM_000186.3 | [1:196,652,043-196,747,504](https://genome.ucsc.edu/cgi-bin/hgTracks?db=hg38&position=chr1:196652043-196747504&dgv=pack&knownGene=pack&omimGene=pack) |
| 15 | *CLCN5* | NM_001127899.3 | [X:49,922,596-50,099,230](https://genome.ucsc.edu/cgi-bin/hgTracks?db=hg38&position=chrX:49922596-50099230&dgv=pack&knownGene=pack&omimGene=pack) |
| 16 | *COL4A1* | NM_001845.5 | [13:110,148,963-110,307,157](https://genome.ucsc.edu/cgi-bin/hgTracks?db=hg38&position=chr13:110148963-110307157&dgv=pack&knownGene=pack&omimGene=pack) |
| 17 | *COL4A3* | NM_000091.4 | [2:227,164,624-227,314,792](https://genome.ucsc.edu/cgi-bin/hgTracks?db=hg38&position=chr2:227164624-227314792&dgv=pack&knownGene=pack&omimGene=pack) |
| 18 | *COL4A4* | NM_000092.4 | [2:226,967,360-227,164,488](https://genome.ucsc.edu/cgi-bin/hgTracks?db=hg38&position=chr2:226967360-227164488&dgv=pack&knownGene=pack&omimGene=pack) |
| 19 | *COL4A5* | NM_033380.2 | [X:108,439,838-108,697,545](https://genome.ucsc.edu/cgi-bin/hgTracks?db=hg38&position=chrX:108439838-108697545&dgv=pack&knownGene=pack&omimGene=pack) |
| 20 | *COQ2* | NM_015697.7 | [4:83,263,824-83,285,134](https://genome.ucsc.edu/cgi-bin/hgTracks?db=hg38&position=chr4:83263824-83285134&dgv=pack&knownGene=pack&omimGene=pack) |
| 21 | *COQ6* | NM_182476.2 | [14:73,949,918-73,963,670](https://genome.ucsc.edu/cgi-bin/hgTracks?db=hg38&position=chr14:73949918-73963670&dgv=pack&knownGene=pack&omimGene=pack) |
| 22 | *COQ7* | NM_016138.4 | [16:19,067,614-19,083,097](https://genome.ucsc.edu/cgi-bin/hgTracks?db=hg38&position=chr16:19067614-19083097&dgv=pack&knownGene=pack&omimGene=pack) |
| 23 | *COQ8B* | NM_024876.3 | [19:40,691,530-40,716,886](https://genome.ucsc.edu/cgi-bin/hgTracks?db=hg38&position=chr19:40691530-40716886&dgv=pack&knownGene=pack&omimGene=pack) |
| 24 | *COQ9* | NM_020312.3 | [16:57,447,479-57,461,270](https://genome.ucsc.edu/cgi-bin/hgTracks?db=hg38&position=chr16:57447479-57461270&dgv=pack&knownGene=pack&omimGene=pack) |
| 25 | *CRB2* | NM_173689.6 | [9:123,354,065-123,380,326](https://genome.ucsc.edu/cgi-bin/hgTracks?db=hg38&position=chr9:123354065-123380326&dgv=pack&knownGene=pack&omimGene=pack) |
| 26 | *CUBN* | NM_001081.3 | [10:16,823,966-17,129,811](https://genome.ucsc.edu/cgi-bin/hgTracks?db=hg38&position=chr10:16823966-17129811&dgv=pack&knownGene=pack&omimGene=pack) |
| 27 | *CYP11B2* | NM_000498.3 | [8:142,910,559-142,917,843](https://genome.ucsc.edu/cgi-bin/hgTracks?db=hg38&position=chr8:142910559-142917843&dgv=pack&knownGene=pack&omimGene=pack) |
| 28 | *DGKE* | NM_003647.2 | [17:56,834,151-56,869,567](https://genome.ucsc.edu/cgi-bin/hgTracks?db=hg38&position=chr17:56834151-56869567&dgv=pack&knownGene=pack&omimGene=pack) |
| 29 | *E2F3* | NM_001949.7 | [6:20,401,879-20,493,714](https://genome.ucsc.edu/cgi-bin/hgTracks?db=hg38&position=chr6:20401879-20493714&dgv=pack&knownGene=pack&omimGene=pack) |
| 30 | *EMP2* | NM_001424.5 | [16:10,528,422-10,580,598](https://genome.ucsc.edu/cgi-bin/hgTracks?db=hg38&position=chr16:10528422-10580598&dgv=pack&knownGene=pack&omimGene=pack) |
| 31 | *FAT1* | NM_005245.3 | [4:186,587,794-186,726,696](https://genome.ucsc.edu/cgi-bin/hgTracks?db=hg38&position=chr4:186587794-186726696&dgv=pack&knownGene=pack&omimGene=pack) |
| 32 | *GAPVD1* | [NM_001282680.3](http://www.ncbi.nlm.nih.gov/nuccore/NM_001282680.3) | [9:125,261,826-125,367,207](https://genome.ucsc.edu/cgi-bin/hgTracks?db=hg38&position=chr9:125261826-125367207&dgv=pack&knownGene=pack&omimGene=pack) |
| 33 | *GLA* | NM_000169.2 | [X:101,397,803-101,407,925](https://genome.ucsc.edu/cgi-bin/hgTracks?db=hg38&position=chrX:101397803-101407925&dgv=pack&knownGene=pack&omimGene=pack) |
| 34 | *HP* | [NM_005143.5](http://www.ncbi.nlm.nih.gov/nuccore/NM_005143.5) | [16:72,054,505-72,061,055](https://genome.ucsc.edu/cgi-bin/hgTracks?db=hg38&position=chr16:72054505-72061055&dgv=pack&knownGene=pack&omimGene=pack) |
| 35 | *INF2* | NM_022489.3 | [14:104,681,133-104,722,535](https://genome.ucsc.edu/cgi-bin/hgTracks?db=hg38&position=chr14:104681133-104722535&dgv=pack&knownGene=pack&omimGene=pack) |
| 36 | *ITGA3* | NM_002204.3 | [17:50,056,110-50,090,481](https://genome.ucsc.edu/cgi-bin/hgTracks?db=hg38&position=chr17:50056110-50090481&dgv=pack&knownGene=pack&omimGene=pack) |
| 37 | *ITGB4* | NM_000213.4 | [17:75,721,459-75,757,818](https://genome.ucsc.edu/cgi-bin/hgTracks?db=hg38&position=chr17:75721459-75757818&dgv=pack&knownGene=pack&omimGene=pack) |
| 38 | *ITGB8* | [NM_002214.3](http://www.ncbi.nlm.nih.gov/nuccore/NM_002214.3) | [7:20,329,766-20,415,754](https://genome.ucsc.edu/cgi-bin/hgTracks?db=hg38&position=chr7:20329766-20415754&dgv=pack&knownGene=pack&omimGene=pack) |
| 39 | *KANK1* | NM_015158.3 | [9:470,295-746,103](https://genome.ucsc.edu/cgi-bin/hgTracks?db=hg38&position=chr9:470295-746103&dgv=pack&knownGene=pack&omimGene=pack) |
| 40 | *KANK2* | NM_015493 | [19:11,164,270-11,197,865](https://genome.ucsc.edu/cgi-bin/hgTracks?db=hg38&position=chr19:11164270-11197865&dgv=pack&knownGene=pack&omimGene=pack) |
| 41 | *KANK4* | NM_181712.4 | [1:62,236,165-62,319,434](https://genome.ucsc.edu/cgi-bin/hgTracks?db=hg38&position=chr1:62236165-62319434&dgv=pack&knownGene=pack&omimGene=pack) |
| 42 | *KIRREL2* | [NM_199180.4](http://www.ncbi.nlm.nih.gov/nuccore/NM_199180.4) | [19:35,851,399-35,867,136](https://genome.ucsc.edu/cgi-bin/hgTracks?db=hg38&position=chr19:35851399-35867136&dgv=pack&knownGene=pack&omimGene=pack) |
| 43 | *LAGE3* | [NM_006014.5](http://www.ncbi.nlm.nih.gov/nuccore/NM_006014.5) | [X:154,477,775-154,479,281](https://genome.ucsc.edu/cgi-bin/hgTracks?db=hg38&position=chrX:154477775-154479281&dgv=pack&knownGene=pack&omimGene=pack) |
| 44 | *LAMA5* | [NM_005560.6](http://www.ncbi.nlm.nih.gov/nuccore/NM_005560.6) | [20:62,309,065-62,367,312](https://genome.ucsc.edu/cgi-bin/hgTracks?db=hg38&position=chr20:62309065-62367312&dgv=pack&knownGene=pack&omimGene=pack) |
| 45 | *LAMB2* | NM_002292.3 | [3:49,121,114-49,133,050](https://genome.ucsc.edu/cgi-bin/hgTracks?db=hg38&position=chr3:49121114-49133050&dgv=pack&knownGene=pack&omimGene=pack) |
| 46 | *LMNA* | NM_170707.3 | [1:156,082,573-156,140,081](https://genome.ucsc.edu/cgi-bin/hgTracks?db=hg38&position=chr1:156082573-156140081&dgv=pack&knownGene=pack&omimGene=pack) |
| 47 | *LMX1B* | NM_002316.3 | [9:126,613,928-126,701,032](https://genome.ucsc.edu/cgi-bin/hgTracks?db=hg38&position=chr9:126613928-126701032&dgv=pack&knownGene=pack&omimGene=pack) |
| 48 | *MAFB* | [NM_005461.5](http://www.ncbi.nlm.nih.gov/nuccore/NM_005461.5) | [20:40,685,848-40,689,236](https://genome.ucsc.edu/cgi-bin/hgTracks?db=hg38&position=chr20:40685848-40689236&dgv=pack&knownGene=pack&omimGene=pack) |
| 49 | *MAGI2* | NM_012301.3 | [7:78,017,055-79,453,667](https://genome.ucsc.edu/cgi-bin/hgTracks?db=hg38&position=chr7:78017055-79453667&dgv=pack&knownGene=pack&omimGene=pack) |
| 50 | *MED28* | NM_025205.4 | [4:17,614,641-17,634,105](https://genome.ucsc.edu/cgi-bin/hgTracks?db=hg38&position=chr4:17614641-17634105&dgv=pack&knownGene=pack&omimGene=pack) |
| 51 | *MEFV* | NM_000243.2 | [16:3,242,027-3,256,633](https://genome.ucsc.edu/cgi-bin/hgTracks?db=hg38&position=chr16:3242027-3256633&dgv=pack&knownGene=pack&omimGene=pack) |
| 52 | *MUC1* | NM_001204286.1 | [1:155,185,824-155,192,915](https://genome.ucsc.edu/cgi-bin/hgTracks?db=hg38&position=chr1:155185824-155192915&dgv=pack&knownGene=pack&omimGene=pack) |
| 53 | *MYH9* | NM_002473.5 | [22:36,281,280-36,387,967](https://genome.ucsc.edu/cgi-bin/hgTracks?db=hg38&position=chr22:36281280-36387967&dgv=pack&knownGene=pack&omimGene=pack) |
| 54 | *MYO1E* | NM_004998.3 | [15:59,132,434-59,372,871](https://genome.ucsc.edu/cgi-bin/hgTracks?db=hg38&position=chr15:59132434-59372871&dgv=pack&knownGene=pack&omimGene=pack) |
| 55 | *NEIL1* | NM_001256552.1 | [5:75,347,039-75,357,115](https://genome.ucsc.edu/cgi-bin/hgTracks?db=hg38&position=chr15:75347039-75357115&dgv=pack&knownGene=pack&omimGene=pack) |
| 56 | *NPHP4* | NM_015102.4 | [1:5,862,811-5,992,425](https://genome.ucsc.edu/cgi-bin/hgTracks?db=hg38&position=chr1:5862811-5992425&dgv=pack&knownGene=pack&omimGene=pack) |
| 57 | *NPHS1* | NM_004646.3 | [19:35,825,372-35,852,504](https://genome.ucsc.edu/cgi-bin/hgTracks?db=hg38&position=chr19:35825372-35852504&dgv=pack&knownGene=pack&omimGene=pack) |
| 58 | *NPHS2* | NM_014625.3 | [1:179,550,539-179,575,948](https://genome.ucsc.edu/cgi-bin/hgTracks?db=hg38&position=chr1:179550539-179575948&dgv=pack&knownGene=pack&omimGene=pack) |
| 59 | *NUP107* | NM_020401.3 | [12:68,686,978-68,745,809](https://genome.ucsc.edu/cgi-bin/hgTracks?db=hg38&position=chr12:68686978-68745809&dgv=pack&knownGene=pack&omimGene=pack) |
| 60 | *NUP133* | [NM_018230.3](http://www.ncbi.nlm.nih.gov/nuccore/NM_018230.3) | [1:229,440,259-229,508,341](https://genome.ucsc.edu/cgi-bin/hgTracks?db=hg38&position=chr1:229440259-229508341&dgv=pack&knownGene=pack&omimGene=pack) |
| 61 | *NUP160* | [NM_015231.3](http://www.ncbi.nlm.nih.gov/nuccore/NM_015231.3) | [11:47,778,118-47,848,544](https://genome.ucsc.edu/cgi-bin/hgTracks?db=hg38&position=chr11:47778118-47848544&dgv=pack&knownGene=pack&omimGene=pack) |
| 62 | *NUP205* | NM_015135.2 | [7:135,557,917-135,648,753](https://genome.ucsc.edu/cgi-bin/hgTracks?db=hg38&position=chr7:135557917-135648753&dgv=pack&knownGene=pack&omimGene=pack) |
| 63 | *NUP85* | [NM_024844.5](http://www.ncbi.nlm.nih.gov/nuccore/NM_024844.5) | [17:75,205,679-75,235,758](https://genome.ucsc.edu/cgi-bin/hgTracks?db=hg38&position=chr17:75205679-75235758&dgv=pack&knownGene=pack&omimGene=pack) |
| 64 | *NUP93* | NM_014669.4 | [16:56,730,129-56,850,286](https://genome.ucsc.edu/cgi-bin/hgTracks?db=hg38&position=chr16:56730129-56850286&dgv=pack&knownGene=pack&omimGene=pack) |
| 65 | *NXF5* | NM_032946.2 | [X:101,832,112-101,857,577](https://genome.ucsc.edu/cgi-bin/hgTracks?db=hg38&position=chrX:101832112-101857577&dgv=pack&knownGene=pack&omimGene=pack) |
| 66 | *OCRL* | NM_000276.3 | [X:129,540,259-129,592,556](https://genome.ucsc.edu/cgi-bin/hgTracks?db=hg38&position=chrX:129540259-129592556&dgv=pack&knownGene=pack&omimGene=pack) |
| 67 | *OSGEP* | [NM_017807.4](http://www.ncbi.nlm.nih.gov/nuccore/NM_017807.4) | [14:20,446,401-20,454,812](https://genome.ucsc.edu/cgi-bin/hgTracks?db=hg38&position=chr14:20446401-20454812&dgv=pack&knownGene=pack&omimGene=pack) |
| 68 | *PAX2* | NM_003987.4 | [10:100,735,396-100,829,944](https://genome.ucsc.edu/cgi-bin/hgTracks?db=hg38&position=chr10:100735396-100829944&dgv=pack&knownGene=pack&omimGene=pack) |
| 69 | *PDSS2* | NM_020381.3 | [6:107,152,562-107,459,564](https://genome.ucsc.edu/cgi-bin/hgTracks?db=hg38&position=chr6:107152562-107459564&dgv=pack&knownGene=pack&omimGene=pack) |
| 70 | *PIK3C2A* | [NM_002645.4](http://www.ncbi.nlm.nih.gov/nuccore/NM_002645.4) | [11:17,086,575-17,207,986](https://genome.ucsc.edu/cgi-bin/hgTracks?db=hg38&position=chr11:17086575-17207986&dgv=pack&knownGene=pack&omimGene=pack) |
| 71 | *PLCE1* | NM_016341.3 | [10:93,993,931-94,332,823](https://genome.ucsc.edu/cgi-bin/hgTracks?db=hg38&position=chr10:93993931-94332823&dgv=pack&knownGene=pack&omimGene=pack) |
| 72 | *PMM2* | NM_000303.2 | [16:8,797,839-8,849,325](https://genome.ucsc.edu/cgi-bin/hgTracks?db=hg38&position=chr16:8797839-8849325&dgv=pack&knownGene=pack&omimGene=pack) |
| 73 | *PODXL* | NM_005397.3 | [7:131,500,271-131,556,628](https://genome.ucsc.edu/cgi-bin/hgTracks?db=hg38&position=chr7:131500271-131556628&dgv=pack&knownGene=pack&omimGene=pack) |
| 74 | *PRDM15* | [NM_001040424.3](http://www.ncbi.nlm.nih.gov/nuccore/NM_001040424.3) | [21:41,798,225-41,879,344](https://genome.ucsc.edu/cgi-bin/hgTracks?db=hg38&position=chr21:41798225-41879344&dgv=pack&knownGene=pack&omimGene=pack) |
| 75 | *PTPRO* | NM_030667.2 | [12:15,322,508-15,598,331](https://genome.ucsc.edu/cgi-bin/hgTracks?db=hg38&position=chr12:15322508-15598331&dgv=pack&knownGene=pack&omimGene=pack) |
| 76 | *SCARB2* | NM_005506.3 | [4:76,158,737-76,234,532](https://genome.ucsc.edu/cgi-bin/hgTracks?db=hg38&position=chr4:76158737-76234532&dgv=pack&knownGene=pack&omimGene=pack) |
| 77 | *SEMA3A* | [NM_006080.3](http://www.ncbi.nlm.nih.gov/nuccore/NM_006080.3) | [7:83,955,777-84,492,725](https://genome.ucsc.edu/cgi-bin/hgTracks?db=hg38&position=chr7:83955777-84492725&dgv=pack&knownGene=pack&omimGene=pack) |
| 78 | *SEMA3G* | [NM_020163.3](http://www.ncbi.nlm.nih.gov/nuccore/NM_020163.3) | [3: 52,433,035-52,445,103](https://www.ensembl.org/Homo_sapiens/Location/View?db=core;g=ENSG00000010319;r=3:52433035-52445103) |
| 79 | *SGPL1* | [NM_003901.4](http://www.ncbi.nlm.nih.gov/nuccore/NM_003901.4) | [10:70,815,948-70,881,184](https://genome.ucsc.edu/cgi-bin/hgTracks?db=hg38&position=chr10:70815948-70881184&dgv=pack&knownGene=pack&omimGene=pack) |
| 80 | *SMAD9* | [NM_001127217.3](http://www.ncbi.nlm.nih.gov/nuccore/NM_001127217.3) | [13:36,844,831-36,920,854](https://genome.ucsc.edu/cgi-bin/hgTracks?db=hg38&position=chr13:36844831-36920854&dgv=pack&knownGene=pack&omimGene=pack) |
| 81 | *SMARCAL1* | NM_014140.3 | [2:216,412,484-216,483,053](https://genome.ucsc.edu/cgi-bin/hgTracks?db=hg38&position=chr2:216412484-216483053&dgv=pack&knownGene=pack&omimGene=pack) |
| 82 | *SYNPO* | NM_007286.5 | [5:150,586,010-150,659,207](https://genome.ucsc.edu/cgi-bin/hgTracks?db=hg38&position=chr5:150586010-150659207&dgv=pack&knownGene=pack&omimGene=pack) |
| 83 | *TBC1D8B* | [NM_017752.3](http://www.ncbi.nlm.nih.gov/nuccore/NM_017752.3) | [X:106,802,673-106,876,150](https://genome.ucsc.edu/cgi-bin/hgTracks?db=hg38&position=chrX:106802673-106876150&dgv=pack&knownGene=pack&omimGene=pack) |
| 84 | *TNS2* | [NM_170754.4](http://www.ncbi.nlm.nih.gov/nuccore/NM_170754.4) | [12:53,046,991-53,064,379](https://genome.ucsc.edu/cgi-bin/hgTracks?db=hg38&position=chr12:53046991-53064379&dgv=pack&knownGene=pack&omimGene=pack) |
| 85 | *TP53RK* | [NM_033550.4](http://www.ncbi.nlm.nih.gov/nuccore/NM_033550.4) | [20:46,684,365-46,689,444](https://genome.ucsc.edu/cgi-bin/hgTracks?db=hg38&position=chr20:46684365-46689444&dgv=pack&knownGene=pack&omimGene=pack) |
| 86 | *TPRKB* | [NM_016058.5](http://www.ncbi.nlm.nih.gov/nuccore/NM_016058.5) | [2:73,729,873-73,737,345](https://genome.ucsc.edu/cgi-bin/hgTracks?db=hg38&position=chr2:73729873-73737345&dgv=pack&knownGene=pack&omimGene=pack) |
| 87 | *TRPC6* | NM_004621.5 | [11:101,451,564-101,584,007](https://genome.ucsc.edu/cgi-bin/hgTracks?db=hg38&position=chr11:101451564-101584007&dgv=pack&knownGene=pack&omimGene=pack) |
| 88 | *TTC21B* | NM_024753.4 | [2:165,873,362-165,953,776](https://genome.ucsc.edu/cgi-bin/hgTracks?db=hg38&position=chr2:165873362-165953776&dgv=pack&knownGene=pack&omimGene=pack) |
| 89 | *VIPAS39* | NM_022067.3 | [14:77,426,675-77,457,601](https://genome.ucsc.edu/cgi-bin/hgTracks?db=hg38&position=chr14:77426675-77457601&dgv=pack&knownGene=pack&omimGene=pack) |
| 90 | *VPS33B* | NM_018668.3 | [15:90,998,416-91,022,621](https://genome.ucsc.edu/cgi-bin/hgTracks?db=hg38&position=chr15:90998416-91022621&dgv=pack&knownGene=pack&omimGene=pack) |
| 91 | *WDR73* | NM_032856.3 | [15:84,639,285-84,654,283](https://genome.ucsc.edu/cgi-bin/hgTracks?db=hg38&position=chr15:84639285-84654283&dgv=pack&knownGene=pack&omimGene=pack) |
| 92 | *WT1* | [NM_024426.6](http://www.ncbi.nlm.nih.gov/nuccore/NM_024426.6) | [11:32,387,775-32,435,539](https://genome.ucsc.edu/cgi-bin/hgTracks?db=hg38&position=chr11:32387775-32435539&dgv=pack&knownGene=pack&omimGene=pack) |
| 93 | *XPO5* | NM_020750.2 | [6:43,522,334-43,576,038](https://genome.ucsc.edu/cgi-bin/hgTracks?db=hg38&position=chr6:43522334-43576038&dgv=pack&knownGene=pack&omimGene=pack) |
| 94 | *ZMPSTE24* | NM_005857.4 | [1:40,258,236-40,294,180](https://genome.ucsc.edu/cgi-bin/hgTracks?db=hg38&position=chr1:40258236-40294180&dgv=pack&knownGene=pack&omimGene=pack) |
|  |  |  |  |

**Supplementary Table 2:** ACMG criteria for all detected SNVs in Egyptian SRNS patients

| **Variant** | **Gene** | **Variant** | **Allele No** | **ACMG classification** | | | | | | | | | | | | |
| --- | --- | --- | --- | --- | --- | --- | --- | --- | --- | --- | --- | --- | --- | --- | --- | --- |
|  |  |  |  | PVS1 | PS1 | PS2 | PS3 | PM1 | PM2 | PM3 | PM4 | PM5 | PP1 | PP2 | PP3 | Variant category |
|  |  |  |  |  |  |  |  |  |  |  |  |  |  |  |  |  |
| 1 | *NPHS2* (NM_014625.4) | c.1A>T | 4 (2 families) | + | + | ̶ | ̶ | ̶ | + | + | ̶ | ̶ | + | ̶ | ̶ | Pathogenic |
| 2 |  | c.167del;p.(Glu56GlyfsTer43) | 2 | + | ̶ | ̶ | ̶ | ̶ | + | + | ̶ | ̶ | ̶ | ̶ | ̶ | Pathogenic |
| 3 |  | c.467dup;p.(Leu156PhefsTer11) | 2 | + | ̶ | ̶ | ̶ | ̶ | + | + | ̶ | ̶ | ̶ | ̶ | ̶ | Pathogenic |
| 4 |  | c.502C>T;p.(Arg168Cys) | 2 | ̶ | ̶ | ̶ | + | + | + | + | ̶ | + | ̶ | ̶ | + | Pathogenic |
| 5 |  | c.596dup;p.(Asn199LysfsTer14) | 2 | + | ̶ | ̶ | ̶ | ̶ | + | ̶ | ̶ | ̶ | ̶ | ̶ | ̶ | Likely pathogenic |
| 6 |  | c.890C>T;p.(Ala297Val) | 2 | ̶ | ̶ | ̶ | + | + | + | + | ̶ | ̶ | ̶ | ̶ | + | Pathogenic |
| 7 |  | c.934C>G;p.(Leu312Val) | 2 | ̶ | ̶ | ̶ | ̶ | + | + | ̶ | ̶ | ̶ | ̶ | + | + | Likely pathogenic |
| 8 | *NPHS1* (NM_004646.4) | c.1135C>T:p.(Arg379Trp) | 2 | ̶ | ̶ | ̶ | + | + | + | + | ̶ | ̶ | ̶ | ̶ | ̶ | Likely pathogenic |
| 9 |  | c.2758T>C;p.(Cys920Arg) | 1 | ̶ | ̶ | ̶ | ̶ | + | + | ̶ | ̶ | ̶ | ̶ | ̶ | + | Likely pathogenic |
| 10 |  | c.3478C>T;p.(Arg1160Ter) | 1 | + | ̶ | ̶ | ̶ | ̶ | + | ̶ | ̶ | ̶ | ̶ | ̶ | ̶ | Likely pathogenic |
| 11 | *SMARCAL1* (NM_014140.4) | c.1096+4A>G | 2 | ̶ | ̶ | ̶ | ̶ | ̶ | + | ̶ | ̶ | ̶ | ̶ | ̶ | + | VUS |
| 12 |  | c.1860G>A;p.(Trp620Ter) | 2 | + | ̶ | ̶ | ̶ | ̶ | + | ̶ | ̶ | ̶ | ̶ | ̶ | ̶ | Likely pathogenic |
| 13 | *WT1* (NM_024426.6) | c.700G>C;p.(Gly234Arg) | 1 | ̶ | ̶ | ̶ | ̶ | ̶ | + | ̶ | ̶ | ̶ | ̶ | + | + | VUS |
| 14 |  | c.1447+5G>A | 1 | ̶ | ̶ | + | ̶ | ̶ | + | ̶ | ̶ | ̶ | ̶ | ̶ | + | Likely pathogenic |
| 15 | *ALOX12B* (NM_001139.3) | c.1790C>A;p.(Ala597Glu) | 2 | ̶ | ̶ | ̶ | ̶ | + | + | + | ̶ | ̶ | ̶ | + | + | Likely pathogenic |
| 16 | *CD2AP* (NM_012120.3) | c.902A>T;p.(Lys301Met) | 1 | ̶ | ̶ | ̶ | ̶ | ̶ | + | ̶ | ̶ | ̶ | ̶ | ̶ | + | VUS |
| 17 | *COL4A3* (NM_000091.5) | c.2126-1G>A | 1 | + | ̶ | ̶ | ̶ | ̶ | + | ̶ | ̶ | ̶ | ̶ | ̶ | ̶ | Likely pathogenic |
| 18 | *C12orf57* (NM_138425.4) | c.1A>G | 2 | + | + | ̶ | + | ̶ | + | + | ̶ | ̶ | ̶ | ̶ | ̶ | Pathogenic |
| 19 | *LAMB2* (NM_002292.4) | c.1178_1180del; p.(Phe393del) | 2 | ̶ | ̶ | ̶ | ̶ | ̶ | + | ̶ | + | ̶ | ̶ | ̶ | ̶ | VUS |
| 20 | *LMX1B* (NM_001174147.2) | c.737G>A;p.(Arg246Gln) | 1 | ̶ | ̶ | + | + | + | + | ̶ | ̶ | + | ̶ | + | + | Pathogenic |
| 21 | *MYO1E* (NM_004998.4) | c.1616+1G>C | 2 | + | ̶ | ̶ | ̶ | ̶ | + | ̶ | ̶ | ̶ | ̶ | ̶ | ̶ | Likely pathogenic |
| 22 | *NUP93* (NM_014669.5) | c.554A>G;p.(Tyr185Cys) | 2 | ̶ | ̶ | ̶ | ̶ | ̶ | + | ̶ | ̶ | ̶ | ̶ | ̶ | + | VUS |
| 23 | *PLCE1* (NM_016341.4) | c.2779G>T;p(.Gly927Ter) | 2 | + | ̶ | ̶ | ̶ | ̶ | + | ̶ | ̶ | ̶ | ̶ | ̶ | ̶ | Likely pathogenic |
| 24 | *PODXL* (NM_001018111.3) | c.1101+2T>C | 2 | + | ̶ | ̶ | ̶ | ̶ | + | ̶ | ̶ | ̶ | ̶ | ̶ | ̶ | Likely pathogenic |

**PVS1**, Null variant in a gene where loss of function is a known mechanism of disease; **PS1,** Same amino acid change as an established pathogenic variant; **PS2**, De novo in a patient with phenotype consistency, no family history and both maternity and paternity are confirmed; **PS3**, Well-established functional studies show a deleterious effect**; PM1**, Non-truncating non-synonymous variant is located in a mutational hot spot and/or critical and well-established functional domain; **PM2**, Extremely low frequency in the gnomAD population database; **PM3:** For recessive disorders, detected in trans with a pathogenic variant, or in a homozygous or compound heterozygous state in affected cases; **PM4**, Protein coding length changes as a result of in frame variant, and this variant is not located in a repeat region; **PM5**, Different amino acid change as a known pathogenic variant; **PP1,** co-segregation in multiple family members; **PP2**, Missense variant in a gene with low rate of benign missense mutations and for which missense mutation is a common mechanism of a disease; **PP3**, For a missense or a splicing region variant, computational prediction tools unanimously support a deleterious effect on the gene [26]. Scaled point system for pathogenicity scoring was used as described by Tavtigian et al., 2020 [27].

**Supplementary Table 3:** Genes deleted in family 21 with 16.11P2 deletion syndrome

| **N** | **Gene** | **Ref-Seq** |
| --- | --- | --- |
|  |  |  |
| 1 | *ALDOA* | [NM_001243177.4](http://www.ncbi.nlm.nih.gov/nuccore/NM_001243177.4) |
| 2 | *ASPHD1* | [NM_181718.4](http://www.ncbi.nlm.nih.gov/nuccore/NM_181718.4) |
| 3 | *C16orf54* | [NM_175900.4](http://www.ncbi.nlm.nih.gov/nuccore/NM_175900.4) |
| 4 | *C16orf92* | [NM_001109659.2](http://www.ncbi.nlm.nih.gov/nuccore/NM_001109659.2) |
| 5 | *CDIPT* | [NM_006319.5](http://www.ncbi.nlm.nih.gov/nuccore/NM_006319.5) |
| 6 | *CORO1A* | [NM_007074.4](http://www.ncbi.nlm.nih.gov/nuccore/NM_007074.4) |
| 7 | *DOC2A* | [NM_003586.3](http://www.ncbi.nlm.nih.gov/nuccore/NM_003586.3) |
| 8 | [*ENSG00000285043*](https://varsome.com/gene/hg38/ENSG00000285043) | [NM_001365304.2](http://www.ncbi.nlm.nih.gov/nuccore/NM_001365304.2) |
| 9 | *GDPD3* | [NM_024307.3](http://www.ncbi.nlm.nih.gov/nuccore/NM_024307.3) |
| 10 | *HIRIP3* | [NM_003609.5](http://www.ncbi.nlm.nih.gov/nuccore/NM_003609.5) |
| 11 | *INO80E* | [NM_173618.3](http://www.ncbi.nlm.nih.gov/nuccore/NM_173618.3) |
| 12 | *KCTD13* | [NM_178863.5](http://www.ncbi.nlm.nih.gov/nuccore/NM_178863.5) |
| 13 | *KIF22* | [NM_007317.3](http://www.ncbi.nlm.nih.gov/nuccore/NM_007317.3) |
| 14 | *LOC112694756* | [NM_001365304.2](http://www.ncbi.nlm.nih.gov/nuccore/NM_001365304.2) |
| 15 | *MAPK3* | [NM_002746.3](http://www.ncbi.nlm.nih.gov/nuccore/NM_002746.3) |
| 16 | *MAZ* | [NM_002383.4](http://www.ncbi.nlm.nih.gov/nuccore/NM_002383.4) |
| 17 | *MVP* | [NM_005115.5](http://www.ncbi.nlm.nih.gov/nuccore/NM_005115.5) |
| 18 | *PAGR1* | [NM_024516.4](http://www.ncbi.nlm.nih.gov/nuccore/NM_024516.4) |
| 19 | *PPP4C* | [NM_002720.3](http://www.ncbi.nlm.nih.gov/nuccore/NM_002720.3) |
| 20 | *PRRT2* | [NM_145239.3](http://www.ncbi.nlm.nih.gov/nuccore/NM_145239.3) |
| 21 | *QPRT* | [NM_014298.6](http://www.ncbi.nlm.nih.gov/nuccore/NM_014298.6) |
| 22 | *SEZ6L2* | [NM_001243332.2](http://www.ncbi.nlm.nih.gov/nuccore/NM_001243332.2) |
| 23 | *SPN* | [NM_003123.6](http://www.ncbi.nlm.nih.gov/nuccore/NM_003123.6) |
| 24 | *TAOK2* | [NM_016151.4](http://www.ncbi.nlm.nih.gov/nuccore/NM_016151.4) |
| 25 | *TBX6* | [NM_004608.4](http://www.ncbi.nlm.nih.gov/nuccore/NM_004608.4) |
| 26 | *TLCD3B* | [NM_031478.6](http://www.ncbi.nlm.nih.gov/nuccore/NM_031478.6) |
| 27 | *TMEM219* | [NM_001083613.2](http://www.ncbi.nlm.nih.gov/nuccore/NM_001083613.2) |
| 28 | *YPEL3* | [NM_031477.5](http://www.ncbi.nlm.nih.gov/nuccore/NM_031477.5) |
| 29 | *ZG16* | [NM_152338.4](http://www.ncbi.nlm.nih.gov/nuccore/NM_152338.4) |

**Supplementary Table 4:** Evaluation of the effects of protein amino acid changes in detected missense variants on protein structure and function.

| **Gene** | **Variants** | **Structural scores** | | | **Conservation score** | **Structure** |
| --- | --- | --- | --- | --- | --- | --- |
|  |  | MAESTRO web | | I-mutant  DDG Prediction | PhyloP100 |  |
|  |  | ΔΔGpred | cpred. |  |  |  |
| ***LMX1B*** | **p.(Arg246Gln)** | 0.200 | 0.849 | -2.48 | 9.415 | 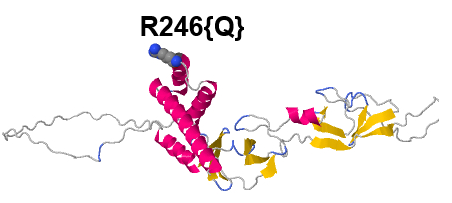  AF-O60663, Model Confidence: Very high (pLDDT > 94.95) |
| ***NPHS2*** | **p.(Leu312Val)** | -0.262 | 0.931 | -1.56 | 2.386 | 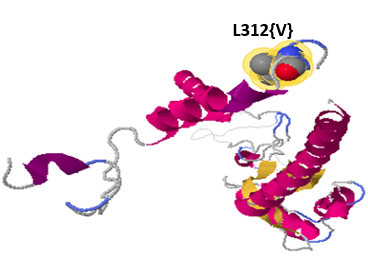  AF-Q9NP85, Model Confidence: Very high (pLDDT > 98.04) |
| ***NPHS2*** | **p.(Ala297Val)** | 0.294 | 0.862 | -0.43 | 4.704 | 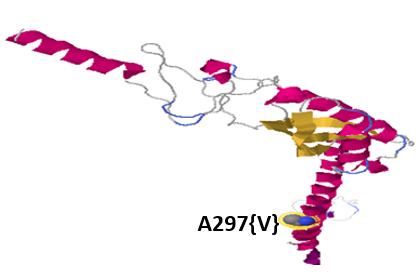  AF-Q9NP85, Model Confidence: Very high (pLDDT > 98.04) |
| ***NPHS2*** | **p.(Arg168Cys)** | 1.011 | 0.830 | -0.84 | 5.709 | 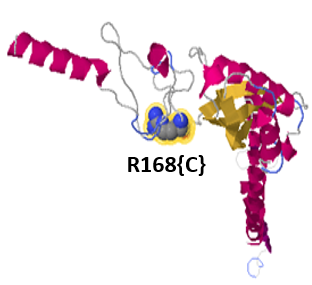  AF-Q9NP85, Model Confidence: Very high (pLDDT > 98.04) |
| ***NUP93*** | **p.(Tyr185Cys)** | 0.209 | 0.949 | -2.48 | 7.782 | 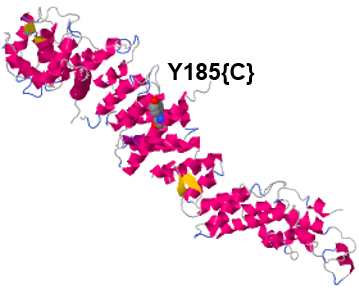  PDB.Id: 7MW0, Method: X-ray, Resolution: 2.00Å |
| ***NPHS1*** | **p.(Arg379Trp)** | 0.025 | 0.824 | -0.09 | 1.678 | 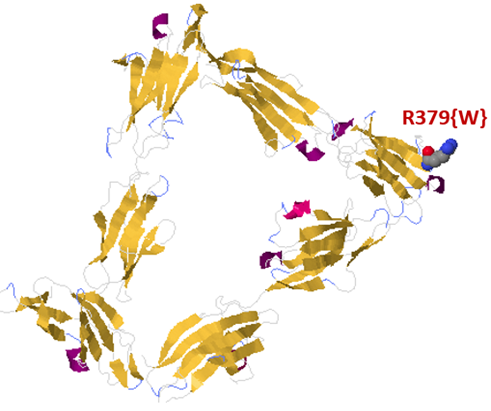  AF-O60500-F1, Model Confidence: Very high (pLDDT>93.37) |
| ***NPHS1*** | **p.(Cys920Arg)** | 0.225 | 0.843 | 0.18 | 3.781 | 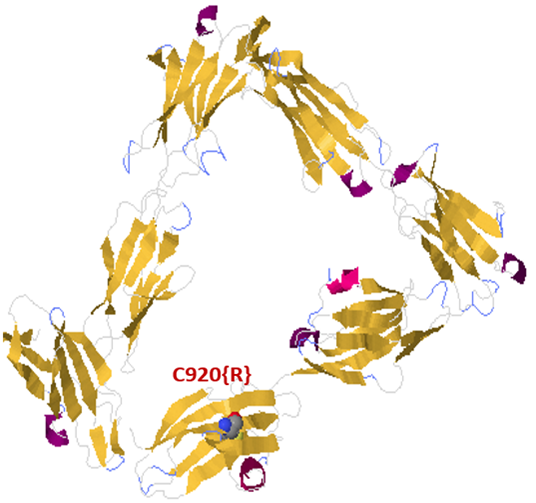  AF-O60500-F1, Model Confidence: Very high (pLDDT>93.37) |
| ***ALOX12B*** | **p.(Ala597Glu)** | 0.421 | 0.949 | -0.56 | 6.074 | 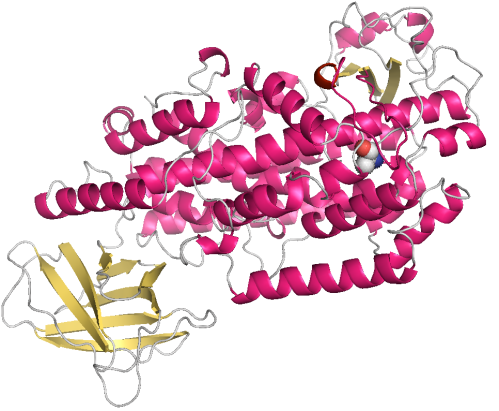  **A597{G}**  AF-O75342-F1, Model Confidence: Very high (pLDDT>96.85) |
| ***CD2AP*** | **p.(Lys301Met)** | 0.015 | 0.904 | -0.668 | 7.073 | 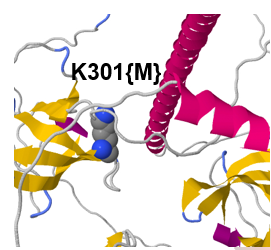  AF-Q9Y5K6-F1, Model Confidence: Very high (pLDDT >91.13) |
| ***WT1*** | **p.(Glu234Arg)** | -0.080 | 0.77 | -0.43 | 4.428 | **G234{R}**  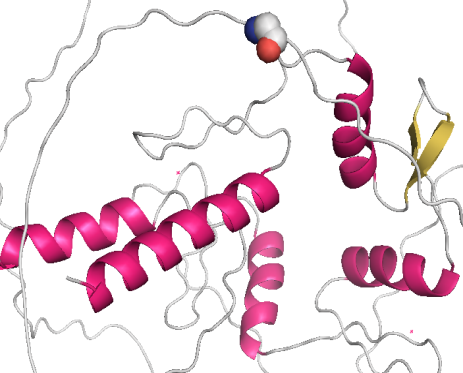  AF-P19544-F1, Model Confidence: Very low (pLDDT >35.71) |

For MAESTRO web classification: ΔΔGpred: total predicted change in stability (kcal/mol). ΔΔGpred.>0.0 indicates a destabilizing variant. cpred. confidence estimation, given as value between 0.0 (not reliable) and 1.0 (highly reliable). For I-mutant server classification (SVM2): DDG<0: Decreased Stability, DDG>0: Increased Stability. PhyloP100 score evaluates the conservation of the affected base in 100 vertebrate species, a positive value indicates conservation.

The data reported here quantifies structural and evolutionary impacts of these variants using MAESTRO web and I-mutant, with positive ΔΔGpred values. Most variants show decreased stability (negative I-mutant DDG), supporting their potential disease relevance. First, regarding to Stability, destabilizing variants (ΔΔGpred <0): *LMX1B* p.(Arg246Gln) (ΔΔGpred = -2.48) likely disrupts DNA-binding helices, while *NPHS2* p.(Leu312Val) (ΔΔGpred = -1.56) may impair podocin stability. Also, rare stabilizing variants (ΔΔGpred >0): *NPHS2* p.(Arg168Cys) (ΔΔGpred = +1.01) could induce abnormal rigidity, altering functional dynamics. Second, regarding conservation (PhyloP100): variants have high conservation scores (>7, e.g., *LMX1B* p.(Arg246Gln): 9.415) highlight evolutionarily conserved residues, suggesting critical roles. Substitutions here (e.g., Arg→Gln) may perturb conserved functional motifs. Also, low scores (e.g., *NPHS1* p.(Arg379Trp): 1.678) suggest lower evolutionary constraint but may still impact non-conserved structural regions. Finally, regarding function, variants in *NPHS2/NPHS1* destabilize structures (negative ΔΔGpred) and cluster in functional domains, aligning with their roles in nephrotic syndrome. *LMX1B* p.(Arg246Gln) (high conservation, destabilizing) likely disrupts DNA-binding helices, explaining their link to limb/kidney malformations. *WT1* p.(Glu234Arg), despite low model confidence, localizes to a zinc-finger domain; experimental validation is needed to confirm DNA-binding defects. Overall, the combination of Figure 2 and Supplementary Table 4 provides a multi-faceted view of these missense variants, linking structural changes to predicted functional impacts. The destabilizing effects seen in most variants, particularly those with high conservation scores, suggest that they may contribute to disease phenotypes by impairing protein stability. These findings underscore the importance of integrating structural modeling with computational predictions to assess the pathogenicity of genetic variants.
