# Supplementary material for: Whole genome sequencing identifies monogenic disease in 56.1% of families with early-onset steroid-resistant nephrotic syndrome
Source: Hum Genet. 2025 May 22;144(7):727–40. doi: 10.1007/s00439-025-02752-y (PMC12222417; doi:10.1007/s00439-025-02752-y)
Supplement: Supplementary file 2 — Supplementary file2 (DOCX 222 KB) [file 439_2025_2752_MOESM2_ESM.docx]

**Whole genome sequencing identifies monogenic disease in 56.1% of families with steroid-resistant nephrotic syndrome**

**Running title: Genomics of SRNS in Egypt**

**Neveen A. Soliman^1,2,3^*, Mohamed A. Elmonem^2,4^, Ahmed F. El-Sayed^2,5^, Eman Ramadan^2,6^, Ahmed M. Badr^1,3^, Fatma M. Atia^1,3^, Rasha Helmy^1,3^, May O. Amer^2^, Ahmed Abd El-Raouf^2^, Fadya M. El-Garhy^2^, Omnia M. Abdel-Haseb^2^, Tokka M. Hassan^2^, Yasmeen K. Farouk^2^, Ahmed El-Hosseiny^2,7^, Usama Bakry^2^, Asmaa Ali^2^, Sheri Saleeb^2^, Tasnim A. Ghanim^2^, Mahynour Albarbary^2^, Ahmed Elmahy^2^, Tarek Elnagdy^2^, Amira Ragheb^2^, Wael A. Hassan^2^, Ahmed Moustafa^2,7^, Khaled Amer^2^***

^1^ Department of Pediatrics, Center for Pediatric Nephrology and Transplantation (CPNT), Faculty of Medicine, Cairo University, Cairo, Egypt

^2^ Egypt Center for Research and Regenerative Medicine (ECRRM), Cairo, Egypt

^3^ EGORD, Egyptian group of Orphan Renal Diseases, Cairo, Egypt

^4^ Department of Clinical and Chemical Pathology, Faculty of Medicine, Cairo University, Cairo, Egypt

^5^ Department of Microbial Genetics, Biotechnology Research Institute, National Research Centre (NRC), Giza, Egypt

^6^ Pharmacology and Biochemistry Department, Faculty of Pharmacy, the British University in Egypt, Cairo, Egypt

^7^ Department of Biology, American University in Cairo, New Cairo, Egypt

**Corresponding authors**

Prof. Dr. Neveen A. Soliman, [nsoliman@kasralainy.edu.eg](mailto:nsoliman@kasralainy.edu.eg); [neveenase@yahoo.com](mailto:neveenase@yahoo.com), ORCID: [0000-0002-8942-1973](https://orcid.org/0000-0002-8942-1973)

Prof. Dr. Khaled Amer, [khaled.amer@ecrrm.ac.eg](mailto:khaled.amer@ecrrm.ac.eg); [dramertx@gmail.com](mailto:dramertx@gmail.com), ORCID 0000-0001-6896-9449

**Supplementary Tables and Figures**

**Supplementary Table 1: SRNS related genes**

| N | Gene | Ref-Seq |
| --- | --- | --- |
|  |  |  |
| 1 | ACTN4 | NM_004924.5 |
| 2 | ALG1 | NM_019109.4 |
| 3 | ALMS1 | NM_015120.4 |
| 4 | ALOX12B | [NM_001139.3](http://www.ncbi.nlm.nih.gov/nuccore/NM_001139.3) |
| 5 | ANKFY1 | [NM_001330063.2](http://www.ncbi.nlm.nih.gov/nuccore/NM_001330063.2) |
| 6 | ANKS6 | NM_173551.4 |
| 7 | ANLN | NM_018685.4 |
| 8 | APOL1 | [NM_003661.4](http://www.ncbi.nlm.nih.gov/nuccore/NM_003661.4) |
| 9 | ARHGAP24 | NM_001025616.2 |
| 10 | ARHGDIA | NM_001185077.2 |
| 11 | AVIL | [NM_006576.4](http://www.ncbi.nlm.nih.gov/nuccore/NM_006576.4) |
| 12 | CD151 | NM_004357.4 |
| 13 | CD2AP | NM_012120.2 |
| 14 | CFH | NM_000186.3 |
| 15 | CLCN5 | NM_001127899.3 |
| 16 | COL4A1 | NM_001845.5 |
| 17 | COL4A3 | NM_000091.4 |
| 18 | COL4A4 | NM_000092.4 |
| 19 | COL4A5 | NM_033380.2 |
| 20 | COQ2 | NM_015697.7 |
| 21 | COQ6 | NM_182476.2 |
| 22 | COQ7 | NM_016138.4 |
| 23 | COQ8B | NM_024876.3 |
| 24 | COQ9 | NM_020312.3 |
| 25 | CRB2 | NM_173689.6 |
| 26 | CUBN | NM_001081.3 |
| 27 | CYP11B2 | NM_000498.3 |
| 28 | DGKE | NM_003647.2 |
| 29 | E2F3 | NM_001949.7 |
| 30 | EMP2 | NM_001424.5 |
| 31 | FAT1 | NM_005245.3 |
| 32 | GAPVD1 | [NM_001282680.3](http://www.ncbi.nlm.nih.gov/nuccore/NM_001282680.3) |
| 33 | GLA | NM_000169.2 |
| 34 | HP | [NM_005143.5](http://www.ncbi.nlm.nih.gov/nuccore/NM_005143.5) |
| 35 | INF2 | NM_022489.3 |
| 36 | ITGA3 | NM_002204.3 |
| 37 | ITGB4 | NM_000213.4 |
| 38 | ITGB8 | [NM_002214.3](http://www.ncbi.nlm.nih.gov/nuccore/NM_002214.3) |
| 39 | KANK1 | NM_015158.3 |
| 40 | KANK2 | NM_015493 |
| 41 | KANK4 | NM_181712.4 |
| 42 | KIRREL2 | [NM_199180.4](http://www.ncbi.nlm.nih.gov/nuccore/NM_199180.4) |
| 43 | LAGE3 | [NM_006014.5](http://www.ncbi.nlm.nih.gov/nuccore/NM_006014.5) |
| 44 | LAMA5 | [NM_005560.6](http://www.ncbi.nlm.nih.gov/nuccore/NM_005560.6) |
| 45 | LAMB2 | NM_002292.3 |
| 46 | LMNA | NM_170707.3 |
| 47 | LMX1B | NM_002316.3 |
| 48 | MAFB | [NM_005461.5](http://www.ncbi.nlm.nih.gov/nuccore/NM_005461.5) |
| 49 | MAGI2 | NM_012301.3 |
| 50 | MED28 | NM_025205.4 |
| 51 | MEFV | NM_000243.2 |
| 52 | MUC1 | NM_001204286.1 |
| 53 | MYH9 | NM_002473.5 |
| 54 | MYO1E | NM_004998.3 |
| 55 | NEIL1 | NM_001256552.1 |
| 56 | NPHP4 | NM_015102.4 |
| 57 | NPHS1 | NM_004646.3 |
| 58 | NPHS2 | NM_014625.3 |
| 59 | NUP107 | NM_020401.3 |
| 60 | NUP133 | [NM_018230.3](http://www.ncbi.nlm.nih.gov/nuccore/NM_018230.3) |
| 61 | NUP160 | [-------------------](http://www.ncbi.nlm.nih.gov/nuccore/NM_018230.3) |
| 62 | NUP205 | NM_015135.2 |
| 63 | NUP85 | [NM_024844.5](http://www.ncbi.nlm.nih.gov/nuccore/NM_024844.5) |
| 64 | NUP93 | NM_014669.4 |
| 65 | NXF5 | NM_032946.2 |
| 66 | OCRL | NM_000276.3 |
| 67 | OSGEP | [NM_017807.4](http://www.ncbi.nlm.nih.gov/nuccore/NM_017807.4) |
| 68 | PAX2 | NM_003987.4 |
| 69 | PDSS2 | NM_020381.3 |
| 70 | PIK3C2A | [NM_002645.4](http://www.ncbi.nlm.nih.gov/nuccore/NM_002645.4) |
| 71 | PLCE1 | NM_016341.3 |
| 72 | PMM2 | NM_000303.2 |
| 73 | PODXL | NM_005397.3 |
| 74 | PRDM15 | [NM_001040424.3](http://www.ncbi.nlm.nih.gov/nuccore/NM_001040424.3) |
| 75 | PTPRO | NM_030667.2 |
| 76 | SCARB2 | NM_005506.3 |
| 77 | SEMA3A | [NM_006080.3](http://www.ncbi.nlm.nih.gov/nuccore/NM_006080.3) |
| 78 | SEMA3G | [NM_020163.3](http://www.ncbi.nlm.nih.gov/nuccore/NM_020163.3) |
| 79 | SGPL1 | [NM_003901.4](http://www.ncbi.nlm.nih.gov/nuccore/NM_003901.4) |
| 80 | SMAD9 | [NM_001127217.3](http://www.ncbi.nlm.nih.gov/nuccore/NM_001127217.3) |
| 81 | SMARCAL1 | NM_014140.3 |
| 82 | SYNPO | NM_007286.5 |
| 83 | TBC1D8B | [NM_017752.3](http://www.ncbi.nlm.nih.gov/nuccore/NM_017752.3) |
| 84 | TNS2 | [NM_170754.4](http://www.ncbi.nlm.nih.gov/nuccore/NM_170754.4) |
| 85 | TP53RK | [NM_033550.4](http://www.ncbi.nlm.nih.gov/nuccore/NM_033550.4) |
| 86 | TPRKB | [NM_016058.5](http://www.ncbi.nlm.nih.gov/nuccore/NM_016058.5) |
| 87 | TRPC6 | NM_004621.5 |
| 88 | TTC21B | NM_024753.4 |
| 89 | VIPAS39 | NM_022067.3 |
| 90 | VPS33B | NM_018668.3 |
| 91 | WDR73 | NM_032856.3 |
| 92 | WT1 | NM_024426_449AAs.3 |
| 93 | XPO5 | NM_020750.2 |
| 94 | ZMPSTE24 | NM_005857.4 |
|  |  |  |

**Supplementary Table 2: Genes deleted in family 21 with 16.11P2 deletion syndrome**

| N | Gene | Ref-Seq |
| --- | --- | --- |
|  |  |  |
| 1 | ALDOA | [NM_001243177.4](http://www.ncbi.nlm.nih.gov/nuccore/NM_001243177.4) |
| 2 | ASPHD1 | [NM_181718.4](http://www.ncbi.nlm.nih.gov/nuccore/NM_181718.4) |
| 3 | C16orf54 | [NM_175900.4](http://www.ncbi.nlm.nih.gov/nuccore/NM_175900.4) |
| 4 | C16orf92 | [NM_001109659.2](http://www.ncbi.nlm.nih.gov/nuccore/NM_001109659.2) |
| 5 | CDIPT | [NM_006319.5](http://www.ncbi.nlm.nih.gov/nuccore/NM_006319.5) |
| 6 | CORO1A | [NM_007074.4](http://www.ncbi.nlm.nih.gov/nuccore/NM_007074.4) |
| 7 | DOC2A | [NM_003586.3](http://www.ncbi.nlm.nih.gov/nuccore/NM_003586.3) |
| 8 | [ENSG00000285043](https://varsome.com/gene/hg38/ENSG00000285043) | [NM_001365304.2](http://www.ncbi.nlm.nih.gov/nuccore/NM_001365304.2) |
| 9 | GDPD3 | [NM_024307.3](http://www.ncbi.nlm.nih.gov/nuccore/NM_024307.3) |
| 10 | HIRIP3 | [NM_003609.5](http://www.ncbi.nlm.nih.gov/nuccore/NM_003609.5) |
| 11 | INO80E | [NM_173618.3](http://www.ncbi.nlm.nih.gov/nuccore/NM_173618.3) |
| 12 | KCTD13 | [NM_178863.5](http://www.ncbi.nlm.nih.gov/nuccore/NM_178863.5) |
| 13 | KIF22 | [NM_007317.3](http://www.ncbi.nlm.nih.gov/nuccore/NM_007317.3) |
| 14 | LOC112694756 | [NM_001365304.2](http://www.ncbi.nlm.nih.gov/nuccore/NM_001365304.2) |
| 15 | MAPK3 | [NM_002746.3](http://www.ncbi.nlm.nih.gov/nuccore/NM_002746.3) |
| 16 | MAZ | [NM_002383.4](http://www.ncbi.nlm.nih.gov/nuccore/NM_002383.4) |
| 17 | MVP | [NM_005115.5](http://www.ncbi.nlm.nih.gov/nuccore/NM_005115.5) |
| 18 | PAGR1 | [NM_024516.4](http://www.ncbi.nlm.nih.gov/nuccore/NM_024516.4) |
| 19 | PPP4C | [NM_002720.3](http://www.ncbi.nlm.nih.gov/nuccore/NM_002720.3) |
| 20 | PRRT2 | [NM_145239.3](http://www.ncbi.nlm.nih.gov/nuccore/NM_145239.3) |
| 21 | QPRT | [NM_014298.6](http://www.ncbi.nlm.nih.gov/nuccore/NM_014298.6) |
| 22 | SEZ6L2 | [NM_001243332.2](http://www.ncbi.nlm.nih.gov/nuccore/NM_001243332.2) |
| 23 | SPN | [NM_003123.6](http://www.ncbi.nlm.nih.gov/nuccore/NM_003123.6) |
| 24 | TAOK2 | [NM_016151.4](http://www.ncbi.nlm.nih.gov/nuccore/NM_016151.4) |
| 25 | TBX6 | [NM_004608.4](http://www.ncbi.nlm.nih.gov/nuccore/NM_004608.4) |
| 26 | TLCD3B | [NM_031478.6](http://www.ncbi.nlm.nih.gov/nuccore/NM_031478.6) |
| 27 | TMEM219 | [NM_001083613.2](http://www.ncbi.nlm.nih.gov/nuccore/NM_001083613.2) |
| 28 | YPEL3 | [NM_031477.5](http://www.ncbi.nlm.nih.gov/nuccore/NM_031477.5) |
| 29 | ZG16 | [NM_152338.4](http://www.ncbi.nlm.nih.gov/nuccore/NM_152338.4) |

**
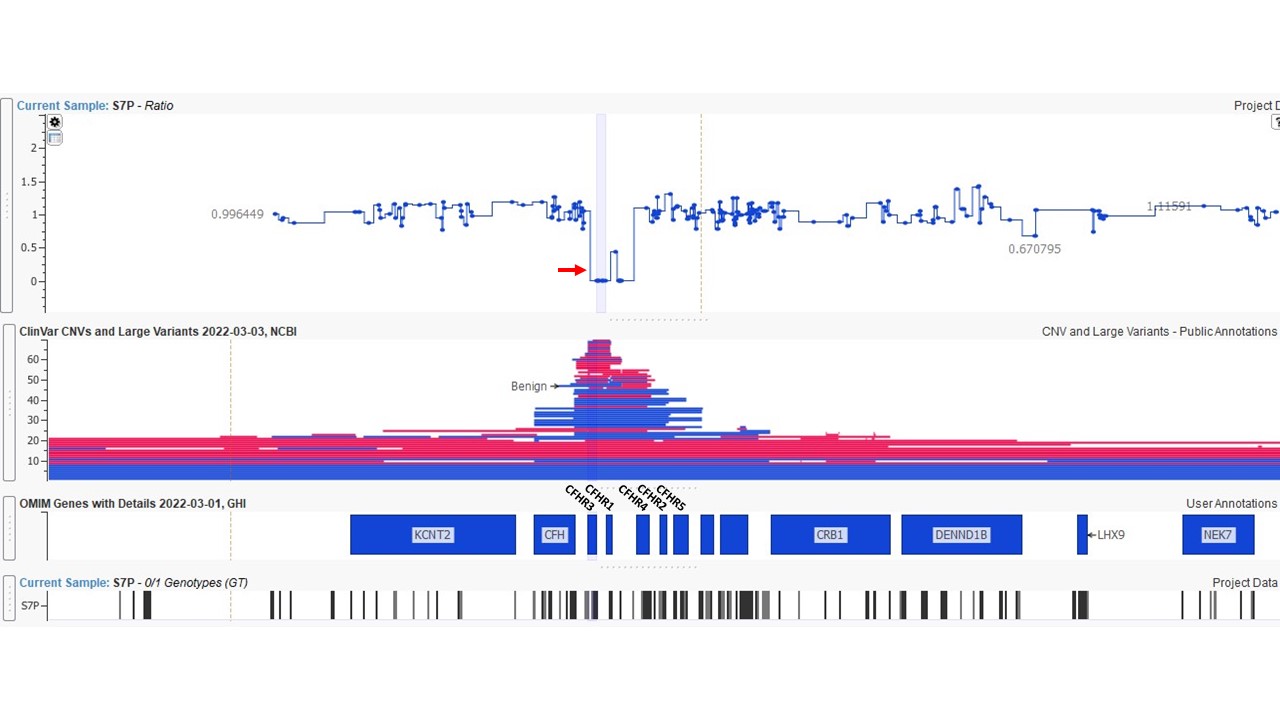
**

**
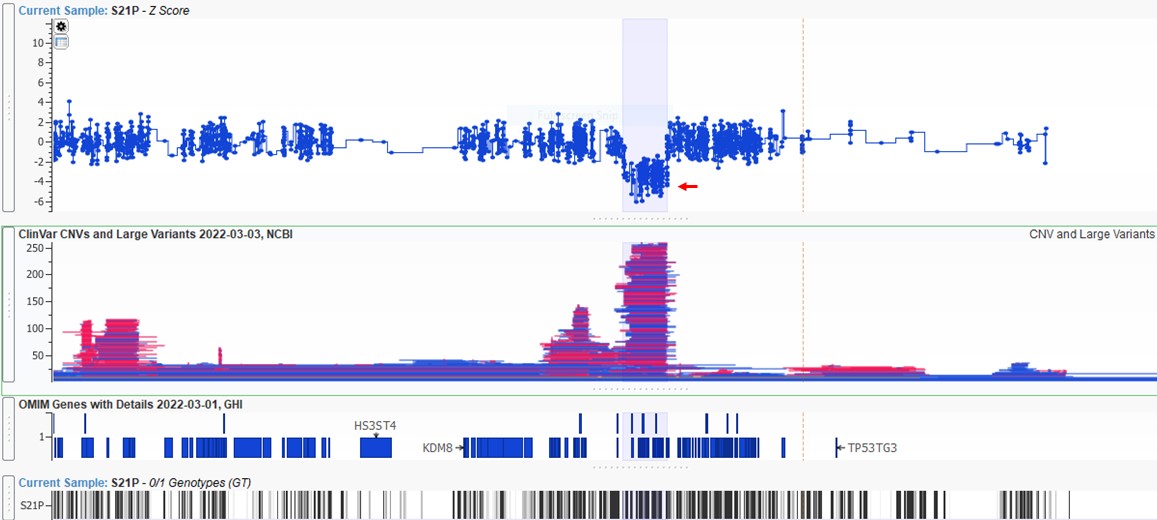
**
